# Tetrazine Carbon Nanotubes for Pretargeted In Vivo “Click‐to‐Release” Bioorthogonal Tumour Imaging

**DOI:** 10.1002/anie.202008012

**Published:** 2020-08-07

**Authors:** He Li, João Conde, Ana Guerreiro, Gonçalo J. L. Bernardes

**Affiliations:** ^1^ Department of Chemistry University of Cambridge Lensfield Road Cambridge CB2 1EW UK; ^2^ Instituto de Medicina Molecular Faculdade de Medicina da Universidade de Lisboa Av. Prof. Egas Moniz 1649-028 Lisboa Portugal

**Keywords:** biorthogonal activation, carbon nanotubes, decaging, fluorogenic, targeted release

## Abstract

The bioorthogonal inverse‐electron‐demand Diels–Alder (IEDDA) cleavage reaction between tetrazine and *trans*‐cyclooctene (TCO) is a powerful way to control the release of bioactive agents and imaging probes. In this study, a pretargeted activation strategy using single‐walled carbon nanotubes (SWCNTs) that bear tetrazines (TZ@SWCNTs) and a TCO‐caged molecule was used to deliver active effector molecules. To optimize a turn‐on signal by using in vivo fluorescence imaging, we developed a new fluorogenic near‐infrared probe that can be activated by bioorthogonal chemistry and image tumours in mice by caging hemicyanine with TCO (tHCA). With our pretargeting strategy, we have shown selective doxorubicin prodrug activation and instantaneous fluorescence imaging in living cells. By combining a tHCA probe and a pretargeted bioorthogonal approach, real‐time, non‐invasive tumour visualization with a high target‐to‐background ratio was achieved in a xenograft mice tumour model. The combined advantages of enhanced stability, kinetics and biocompatibility, and the superior pharmacokinetics of tetrazine‐functionalised SWCNTs could allow application of targeted bioorthogonal decaging approaches with minimal off‐site activation of fluorophore/drug.

## Graphical abstract

**Pretarget then activate**: Functionalization of single‐walled carbon nanotubes (SWCNTs) with tetrazines led to a stable and biocompatible material that undergoes a rapid inverse‐electron‐demand Diels–Alder reaction with *trans*‐cyclooctene caged molecules. This approach enables bioorthogonal activation of responsive drugs or probes in solid tumours in a strictly controlled manner.

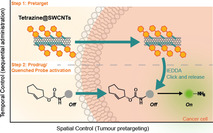

## Introduction

Effective molecular imaging and therapy relies on selective accumulation of a diagnostic probe or therapeutic agent at the site of interest. One such approach is on‐demand activation^1^ in which the potency/activity of the drug/probe is attenuated but which, upon chemical reaction or enzymatic interaction, is converted into its active form at the desired site of action. This method helps spare healthy tissues by preventing adverse and off‐target side effects. Moreover, it allows a precise control of the drug/probe activity and has been employed in the course of pretargeted strategies for imaging and therapy.^1^ Bioorthogonal chemistry is a powerful tool for on‐demand activation. Notably, the inverse‐electron‐demand Diels–Alder (IEDDA) reaction between tetrazine and *trans*‐cyclooctene (TCO) has enormous potential for in vivo bioconjugation by capitalizing on its fast reactivity, even at low concentrations in a complex biological context,^2^ and it is inert to biological functionalities. Furthermore, a “click‐to‐release” bioorthogonal cleavage reaction that enables instantaneous release of a substance from TCO after tetrazine ligation was reported and has potential for prodrug activation.^3^ Prodrug activation with this method was first validated by Robillard and co‐workers who demonstrated efficient and selective activation of a TCO‐caged doxorubicin prodrug^4^ and a monomethyl auristatin E prodrug^5^ from antibody–drug conjugates in tumours. More recently, the same authors have reversed the roles of the TCO and tetrazine.^6^ A TCO reacts with a tetrazine linker that is substituted with methylene‐linked carbamate, which leads to a 1,4‐elimination of the carbamate and release of a secondary amine. Although these examples highlight the potential of bioorthogonal decaging reactions between tetrazine and TCO under complex biological conditions, optimization of the reaction kinetics and pharmacokinetics of the molecules carrying the two complimentary functionalities remains challenging.

**Figure 1:**
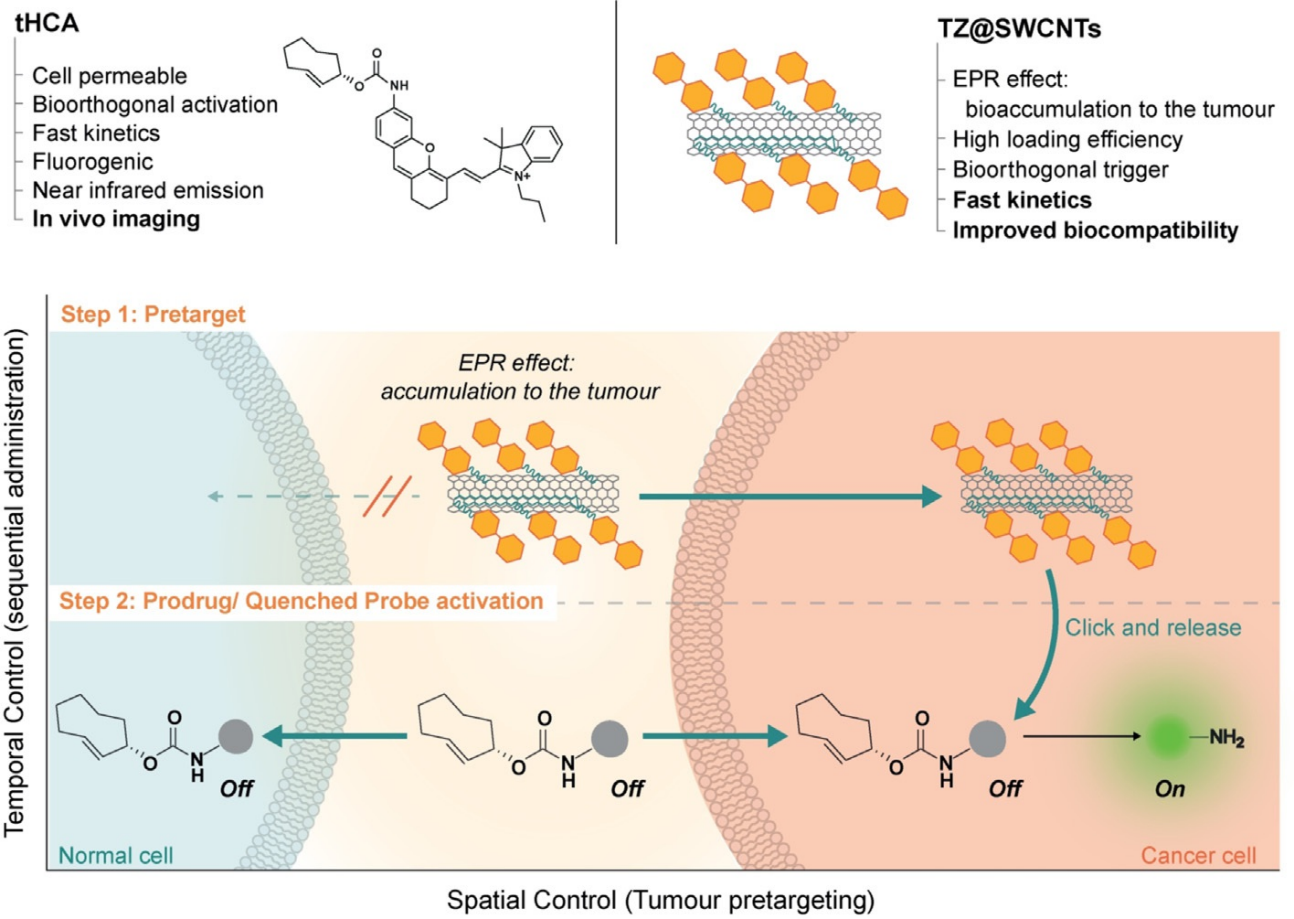
Illustration of the pretargeted strategy for selective activation in cancer cells. The approach takes advantage of the EPR effect‐enabled accumulation of TZ@SWCNTs and the bioorthogonal IEDDA cleavage reaction between tetrazine and TCO to selectively deliver active therapeutic drugs or imaging probes to the tumour.

Herein, we describe a pretargeting strategy that takes advantage of the fast bioorthogonal cleavage between tetrazine and TCO and the tumour‐targeting ability of nanomaterials equipped with bioorthogonal reagents to enable localized enrichment of active drug/probe with tumour specificity and spatiotemporal precision. Single‐walled carbon nanotubes (SWCNTs) have attractive properties; a unique quasi one‐dimensional structure, high loading efficacy, and multivalent effect. SWCNTs also accumulate in tumours^7^ through enhanced permeability and retention (EPR) effects^8^ when systemically injected, which makes them suitable as delivery vehicles for cancer therapy. SWCNTs coated with phospholipid polyethylene glycol (PEG) are stable in vivo with a blood circulation time of approximately 1.2 hours. In addition, SWCNTs also show relatively low uptake by the reticuloendothelial system and near‐complete clearance from the main organs after about 2 months,^9^ which makes these functionalized SWCNTs safe for in vivo applications. SWCNTs are used routinely to diagnose tumours^10^ and therapeutically as carriers to deliver chemotherapeutic drugs,8a, 11 proteins,^12^ plasmid DNA,^13^ and small interfering RNA.^14^ Herein, we present a new strategy in which the bioorthogonal activation of responsive drugs or probes in solid tumours is under strict spatiotemporal control (Figure 1). The strategy comprises two bioorthogonal reagents: tetrazine‐modified SWCNTs (TZ@SWCNTs) and the TCO‐carbamate containing molecules, a chemotherapeutic prodrug or diagnostic probe, which are sequentially administered in two steps. Firstly, TZ@SWCNTs administered systemically will accumulate in tumour tissues as a result of EPR effect. In the second step, the effector molecules whose functions are largely attenuated via TCO protection are systemically applied. The TZ@SWCNTs enriched specifically in tumour sites will serve as a bioorthogonal trigger and activate the effector molecules in situ while sparing normal tissues. This tumour‐pretargeted activation strategy, which explores carbon nanotubes, enables localized enrichment of active drug/probe with spatiotemporal control and selectivity for tumour sites over normal tissues, offering improved effectiveness and safety for tumour imaging and therapy.

Furthermore, we aim to expand the strategy of real‐time targeted fluorescence imaging in vivo, in a non‐destructive way with a high signal‐to‐noise ratio. Fluorescence imaging is a powerful tool in scientific research and medical diagnosis because of its sensitivity and non‐invasiveness, but the limited penetration depth has restrained its further applicability in vivo. A solution to address the problem is to develop and implement fluorophores that emit in the near infrared (NIR) range 700–1000 nm, allowing in‐depth imaging of organisms with minimum background autofluorescence and minimum damage to tissues—a significant advantage over fluorophores that emit in the ultraviolet and visible range. NIR fluorescence imaging is particularly beneficial for image‐guided tumour surgery since it can accurately visualize tumour margins in real time.^15^ Furthermore, an excellent signal‐to‐noise ratio is critical for an effective bioimaging probe. Accordingly, fluorogenic probes that enable turn‐on fluorescence of the reporter in response to a specific trigger are powerful tools for biological imaging and diagnosis in an in vivo context. A TCO‐caged coumarin probe was developed by Chen;3c, 16 however, its emission in the blue region (440–460 nm) limits its use in in vivo applications. Although some tetrazine‐based probes with a turn‐on emission upon IEDDA ligation have been developed, most emit in the UV/Vis range.^17^ There are still a limited number of fluorogenic probes with emission in the NIR range available for chemically controlled illumination in living animals. With this in mind, we seek to develop a fluorogenic bioorthogonal probe that allows for a real‐time targeted fluorescence tumour imaging in vivo.

For our approach, hemicyanine (HCA), a cationic dye, was chosen because it is stable, emits in the NIR, and its optical properties are tuneable through modification of the amine group.^18^ Therefore, HCA was ligated to TCO to give TCO‐carbamate HCA (tHCA). The fluorescence of tHCA can be effectively quenched and restored upon tetrazine‐triggered ligation and liberation. This small molecular probe can diffuse deeply into a tumour and facilitate tumour‐specific imaging when in combination with TZ@SWCNTs.

Overall, our new pretargeted strategy combines SWCNTs and tetrazine/TCO cleavage reaction to 1) enable spatiotemporal control over prodrug activation by integrating the specific targeting property of nanomaterials and the favourable pharmacokinetics and fast clearance of the small effector molecule, and 2) enable real‐time, non‐destructive tumour fluorescence imaging with a high target‐to‐background ratio. We anticipate that this approach, and the reported tetrazine carbon nanotubes and NIR fluorogenic probe, will add significant value to existing targeting strategy for both diagnostic and therapeutic applications.

## Results and Discussion

### Pretargeted imaging with TCO‐AMC carbamate conjugate

To produce TZ@SWCNTs, tetrazine was loaded onto SWCNTs by conjugating methyl tetrazine amine (mTZ) to PEGylated phospholipid (DSPE‐PEG2000) with an *N*‐hydroxysuccinimide terminus to form DSPE‐PEG‐TZ (for synthetic details see the Supporting Information) and then attaching this to SWCNTs by sonication. The resulting TZ@SWCNTs were approximately 200 nm long. This method efficiently loads tetrazine onto SWCNTs, which can reach a loading efficiency of up to 0.24 mmol tetrazine per milligram of SWCNTs. In this study, we used approximately 33 μmol tetrazine per milligram of SWCNTs.

**Figure 2:**
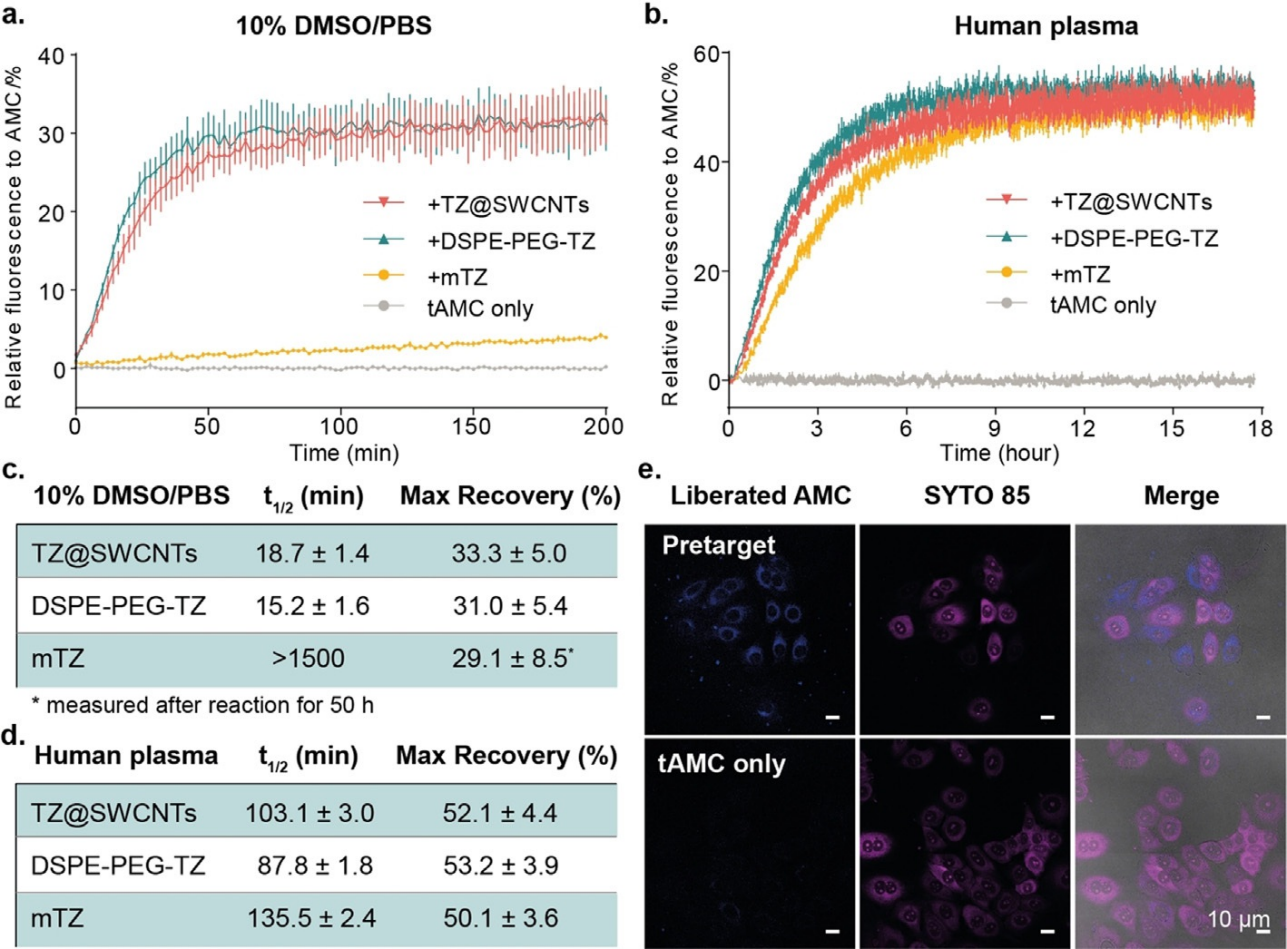
Tetrazine‐responsive tAMC release. a,b) tAMC fluorescence recovery in response to TZ@SWCNTs, DSPE‐PEG‐TZ, or mTZ in 10 % DMSO in PBS (a) and pure human plasma (b) measured by following the appearance of the fluorescence signal of AMC (*λ*_ex_=380 nm, *λ*_em_=445 nm). The data represent the mean ± STD (*n*=3). c,d) Calculated half‐time of the tetrazine‐responsive tAMC decaging reaction and maximum fluorescence recovery in 10 % DMSO in PBS (c) and pure human plasma (d). e) Fluorescence imaging study of the pretargeted tAMC fluorescence activation in MCF‐7 cells. The nuclei were stained with SYTO™ 85 orange; scale bar 10 μm.

To test the feasibility of our pretargeted activation strategy, we firstly demonstrated the design with the reported TCO‐AMC carbamate conjugate (tAMC).^16^ We investigated the kinetics of tAMC liberation in the presence of mTZ, DSPE‐PEG‐TZ, or TZ@SWCNTs (2.5 equiv) in phosphate buffered saline (PBS) with DMSO (10 %) by monitoring the fluorescence recovery (Figure 2 a). AMC release is dramatically faster in response to DSPE‐PEG‐TZ and TZ@SWCNTs relative to the small molecule counterpart mTZ. In the presence of DSPE‐PEG‐TZ and TZ@SWCNTs, tAMC effected over 30 % fluorescence recovery relative to the same concentration of AMC in <50 min, whereas with mTZ the percentage of recovery was 4.2 % and 29 % after 50 minutes and 50 hours, respectively (Figure 2 c). This difference can be explained by the improved solubility from fusing mTZ to the PEG chain. To assess whether the rate acceleration was due to the enhanced cycloaddition reaction or the sequential elimination, we measured the kinetics by following the decrease of tetrazine absorbance at 530 nm. The second‐order rate constants of mTZ and DSPE‐PEG‐TZ toward tAMC are comparable (Supporting Information, Figure S2), which indicates the accelerated tAMC fluorescence recovery in the presence of DSPE‐PEG‐TZ is caused by a faster elimination step. To ensure the reaction could proceed in physiological media, we tested the reaction in pure human plasma. Interestingly, the reaction of DSPE‐PEG‐TZ and TZ@SWCNTs toward tAMC was slower compared to activity in 10 % DMSO/PBS and reached a maximum of 50 % release with a half‐time of 88 and 103 minutes, respectively (Figures 2 b,d). This difference was presumably a result of the increased viscosity of pure human plasma. In contrast, the reaction with mTZ was faster with a half‐time of 135 minutes; the increase is likely due to the improved solubility of mTZ in human serum albumin. Overall, we showed that tetrazine could be loaded onto SWCNTs with high loading efficiency and, importantly, the resulting TZ@SWCNTs demonstrated enhanced decaging reactivity toward tAMC when compared to its small molecule counterpart mTZ.

Subsequently, we studied the pretargeted fluorogenic imaging of tAMC with TZ@SWCNTs on human breast adenocarcinoma (MCF‐7) cells. The cells were pretreated with 50 μm TZ@SWCNTs for 6 hours before being exposed to 10 μm tAMC for another 3 hours. As shown in Figure 2 e, the cells pretreated with TZ@SWCNTs revealed a strong fluorescence signal in cytosol, whereas the cells treated with tAMC only showed minimal background signal. This result indicates that TZ@SWCNTs could effectively target MCF‐7 cells and trigger selective fluorescence restoration of tAMC in living cells.

### Pretargeted prodrug activation

After validation of the pretargeted strategy with tAMC, we explored the application of the strategy in activating prodrugs in living cells. We assessed the biocompatibility of mTZ, DSPE‐PEG‐TZ, and TZ@SWCNTs on three different breast cancer cell lines: MDA‐MB‐231 (triple‐negative breast cancer, negative for HER2 expression), MCF‐7 (breast adenocarcinomas, positive for HER2 expression), and SK‐BR‐3 (breast adenocarcinomas, HER2 over‐expression). Cell viability was evaluated after cell exposure to various concentrations of tetrazines for 24 hours. The three tetrazines exhibited diverse biocompatibilities toward the different cell lines tested. MCF‐7 and SK‐BR‐3 cells exhibited an 80 % cell viability after being exposed to 100 μm TZ@SWCNTs for 24 hours, whereas mTZ and DSPE‐PEG‐TZ showed a much higher toxicity (Figure S7) under the same conditions. This reduced cytotoxicity of TZ@SWCNTs could be a result of the reduced availability of free DSPE‐PEG‐TZ once attached to SWCNTs, suggesting one more advantage of the nanoconstruction in addition to its intracellular and intratumoural transport capability. These results demonstrate that TZ@SWCNTs have improved biocompatibility relative to free mTZ and DSPE‐PEG‐TZ.

**Figure 3:**
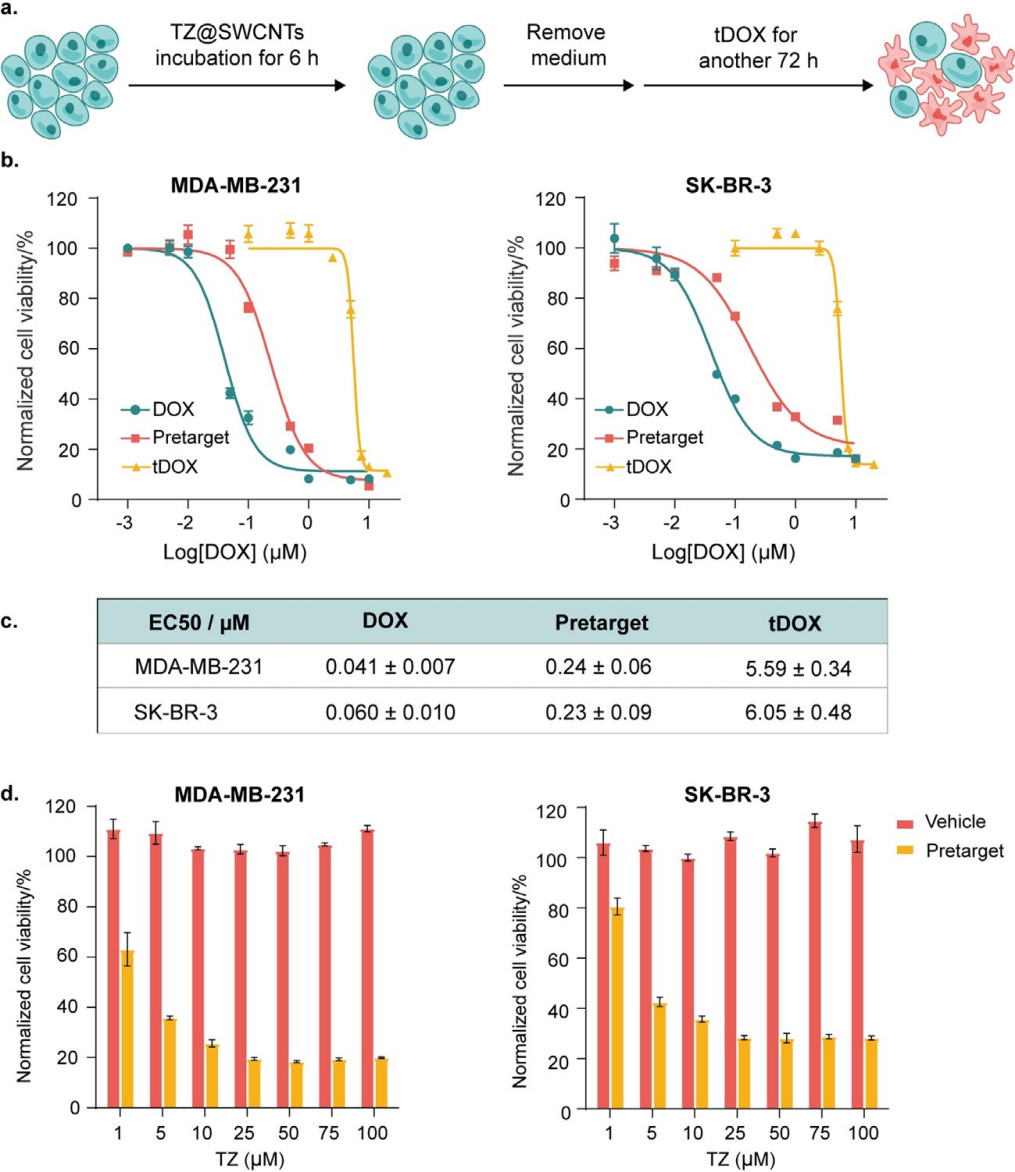
Pretargeted doxorubicin delivery in vitro. a) Flowchart depicting TZ@SWCNTs pretargeting and subsequent tDOX treatment. b) Cytotoxicity of DOX, prodrug tDOX, and pretargeted strategy on MDA‐MB‐231 and SK‐BR‐3 breast cancer cells (*n*=3; error bars represent the STD). Blue and yellow lines indicate cells treated with DOX or tDOX of different concentrations for 72 h. For orange lines, cells were pretreated with TZ@SWCNTs (20 μm) for 6 h before replacing the media to complete medium with various concentration of tDOX for another 72 h. c) Calculated EC_50_ (half‐maximal effective concentration) values for DOX, prodrug tDOX, and pretargeted strategy against MDA‐MB‐231 and SK‐BR‐3 breast cancer cells. d) Cytotoxicity of the vehicle (TZ@SWCNTs, black bar) and pretargeted strategy (grey bar) on MDA‐MB‐231 and SK‐BR‐3 breast cancer cells (*n*=3; error bars represent the STD).

Guided by these results, we explored anticancer doxorubicin (DOX) prodrug activation in living cells with TZ@SWCNTs. The cytotoxicity of TCO‐DOX carbamate (tDOX) prodrug was expected to be attenuated by the chemical modification.^5^ tDOX prodrug was synthesized according to the procedure previously reported.^5^ As illustrated in Figure 3 a, the cytotoxicity of tDOX prodrug, free DOX, and the pretargeted strategy was investigated over 72 hours. For the pretargeted strategy, cells were pretreated with 25 μm TZ@SWCNTs for 6 hours before being exposed to various concentrations of tDOX for another 72 hours. tDOX prodrug displayed significantly reduced cytotoxicity than free DOX with an EC_50_ value of 5.59 μm against MDA‐MB‐231 and 6.05 μm against SK‐BR‐3, which are 138‐ and 100‐fold higher than parent DOX, respectively (Figures 3 b,c). For cells pretargeted with TZ@SWCNTs, the anticancer capability of tDOX was notably restored, presenting a 25‐fold increase in cytotoxicity relative to tDOX alone. The significantly enhanced therapeutic efficiency represents an effective tDOX prodrug activation in cells that contain TZ@SWCNTs. The >25‐fold enhancement of tDOX toxicity in pretargeted cells relative to non‐targeted cells demonstrates improved selectivity and safety of our pretargeted activation strategy over free DOX. Nevertheless, a prodrug approach is not ideal for MCF‐7 cells, which are much less sensitive to DOX.^19^ The EC_50_ of DOX (0.19 μm) was only 43.9 times lower than that of tDOX prodrug (8.3 μm) (Figure S8). For the pretargeted approach, the EC_50_ (1.8 μm) increased 4.6‐fold relative to tDOX alone. These results lead us to suggest that the prodrug activation strategy is more effective for cells with high sensitivity to the parent therapeutic drug. After evaluating the tDOX‐concentration‐dependent cytotoxicity, we assessed the cytotoxicity of 1 μm tDOX in combination with various concentrations of TZ@SWCNTs (Figure 3 d). With subsequent tDOX treatment, TZ@SWCNTs provoked intracellular DOX release and restored therapeutic potency at a concentration as low as 1 μm and reached the highest activation at 25 μm. The trigger TZ@SWCNTs exhibited no obvious inhibition of cell viability up to 100 μm, suggesting the wide selectivity window of the strategy on pretargeted cells over normal cells. These results clearly indicate the efficiency of TZ@SWCNTs to accumulate in cancer cells and liberate active doxorubicin from tDOX prodrug inside live cells, selectively exhibiting anticancer efficacy in pretargeted cells over normal cells.

### Design of an NIR fluorogenic probe tHCA

**Figure 4:**
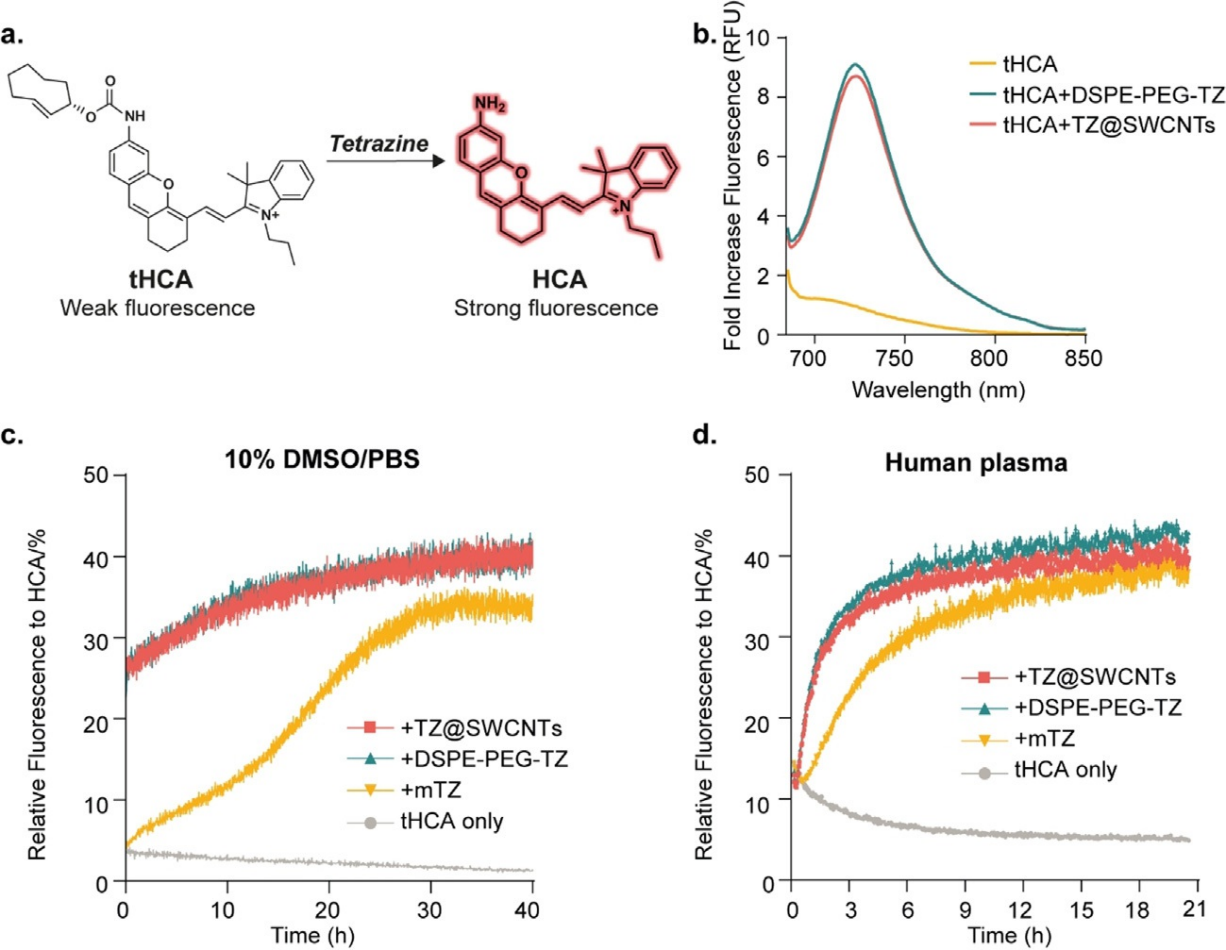
The fluorogenic NIR probe tHCA and its decaging reaction with tetrazine. a) The structure of tHCA and the activation by tetrazine‐mediated cleavage reaction. b) Normalized fluorescence spectra of tHCA (25 μm) before and after reaction with tetrazines (50 μm) in 10 % DMSO/PBS. c,d) Fluorescence recovery profile of tHCA (20 μm) in response to 50 μm TZ@SWCNTs, DSPE‐PEG‐TZ, or mTZ in 10 % DMSO/PBS (c) and pure human plasma (d) (*n*=3; error bars represent the STD) by following the appearance of the fluorescence signal of HCA (*λ*_ex_=665 nm, *λ*_em_=720 nm).

After establishing the pretargeted strategy with the blue fluorescent dye tAMC and the prodrug tDOX, we expanded the strategy to include a fluorogenic NIR tetrazine‐uncaging probe for in vivo applications. The fluorogenic probes only become fluorescent upon activation, enabling the minimization of background signal and omittance of cumbersome washing steps. The NIR emission in the 700–1000 nm window facilitates deep tissue penetration and non‐destructive imaging. To date there are no examples of a fluorogenic NIR probe that can be activated with bioorthogonal control. Therefore, we developed a fluorogenic NIR probe with emission at 720 nm by protecting the amine group on HCA with TCO (Figure 4 a). Starting from a commercially available IR780 iodide, tHCA was obtained in three steps as a dark‐blue solid. The absorption and fluorescence spectra of HCA and tHCA in a pH 7.4 PBS solution containing 10 % DMSO were recorded (Figure S3). HCA shows a maximum absorption at 677 nm; however, upon masking using TCO, the absorbance peak of tHCA exhibited a hypsochromic shift to 656 nm and excitation at 665 nm, resulting in significantly reduced emission at 720 nm (38‐fold less relative to HCA). The shift in absorption and the significant decrease in fluorescent emission indicates that tHCA can be effectively quenched largely by an internal charge‐transfer (ICT) process. Upon addition of TZ@SWCNTs (50 μm), the cleavage reaction was immediate, as detected by a distinct colour change from light blue to dark blue and a remarkable eightfold fluorescence enhancement at 720 nm (Figure 4 b), which demonstrated the fluorogenic nature of the tHCA probe in response to the tetrazine‐mediated cleavage reaction. Induced conversion of tHCA into HCA by DSPE‐PEG‐TZ was further validated by high‐performance liquid chromatography (HPLC; Figure S4). These results confirm that the fluorescence emission of tHCA is effectively quenched by TCO masking and could be restored by tetrazine‐triggered cleavage reaction.

The stability of tHCA in biological context was investigated by HPLC‐PDA (Figure S6). The tHCA was incubated in 20 v/v % human plasma/PBS at 37 °C for 4 hours and 24 hours before the proportion of intact tHCA was analysed by HPLC‐PDA. The results demonstrated that 82 % of tHCA remained intact after 4 hours and 52 % after 24 hours, indicating that tHCA could remain stable during circulation in the blood stream after intravenous injection in mice and, therefore, eligible for systemic in vivo administration. We also evaluated the stability of the three tetrazines in human plasma by measuring their reactivities toward equimolar tHCA. The decaging efficiency of TZ@SWCNTs, DSPE‐PEG‐TZ, and mTZ toward tHCA remained 93, 94, and 77 % respectively after 4 hours of incubation in pure human plasma at 37 °C (Figure S5). These results together confirm that the two components of the strategy, TZ@SWCNTs and tHCA, are stable in physiological media.

Once we verified the fluorogenic nature and the stability of our tetrazine reactive tHCA probe, we investigated the release profile of HCA from tHCA in response to various tetrazines by following the fluorescence emission at 720 nm using a microplate reader. We first tested the reactions in PBS containing 10 % DMSO (Figure 4 c). In the absence of tetrazines, the emission of tHCA (20 μm) remained negligible over the recording period of 40 hours. Once TZ@SWCNTs (50 μm) was added, an immediate fluorescence enhancement of 7.8 times was detected, corresponding to over 25 % liberation of HCA. The fluorescence intensity then gradually increased with time and reached an equilibrium of 41 % recovery at 40 hours. The tHCA release profile triggered by DSPE‐PEG‐TZ mirrored the release in the presence of TZ@SWCNTs. In comparison, the reaction of mTZ with tHCA was much slower with <5 % fluorescence recovery after 60 minutes, 25 % after 20 hours, and a maximum of 35 % recovery after 28 hours.

To further assess the stability and reactivity of the tHCA probe in a biological context, we performed the tetrazine‐mediated tHCA decaging reactions in pure human plasma at 37 °C (Figure 4 d). In the absence of tetrazines, tHCA (20 μm) showed no enhancement of fluorescence intensity in pure human plasma over 24 hours. When incubated with TZ@SWCNTs, DSPE‐PEG‐TZ, and mTZ (50 μm), tHCA reached a maximum 40 % recovery with half‐times of 104, 105, and 211 minutes, respectively. The results were consistent with the fluorescence recovery profiles of tAMC. These results confirm the stability of tHCA and its inducible fluorescence by tetrazine‐mediated decaging reaction in real biological scenarios. These results also highlight the advantage of the fast cleavage reaction between TZ@SWCNTs and tHCA to induce instantaneous fluorescence enhancement.

### Pretargeted fluorogenic NIR imaging in live cells

**Figure 5:**
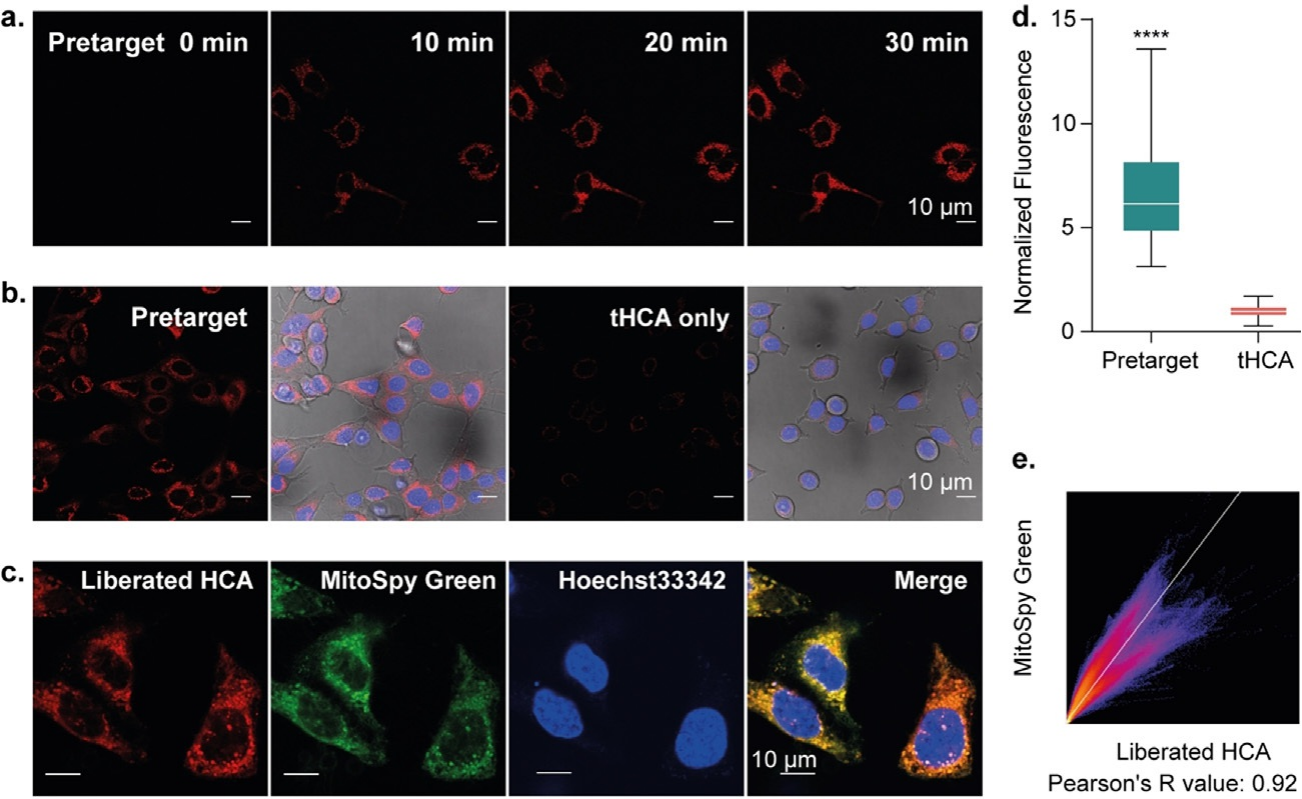
Pretargeted NIR fluorogenic imaging and intracellular colocalization with mitochondria in live cells. a) Real‐time NIR fluorescence imaging in MCF‐7 cells. MCF‐7 cells were pretreated with 50 μm TZ@SWCNTs for 6 h, stained with Hoechst 33342 before adding 1 μm tHCA in PBS for real‐time fluorescence imaging; scale bar=10 μm. b) Comparison of tHCA alone and pretargeted fluorescence imaging at 35 min post tHCA addition. c) Confocal fluorescence images showing the intracellular localization of liberated HCA (red) and the overlap with MitoSpy™ (green); scale bar=10 μm. d) Fluorescence intensity quantification by measuring over 50 cells and represented as box plots. Statistics were calculated with an unpaired t test; ****P<0.0001. e) 2D intensity histogram output of spatial correlation analysis performed with Fiji ImageJ. The Pearson coefficient was calculated to be 0.92.

After verifying the fluorogenic properties, stability, and reactivity of tHCA, we explored the application of our tHCA probe for pretargeted live‐cell imaging. First, we evaluated the biocompatibility of tHCA by measuring the cell viability of MCF‐7 cells incubated with tHCA. The viability of MCF‐7 cells remained over 70 % after incubation with 2.5 μm tHCA for 2 hours and then in complete DMEM for another 72 hours (Figure S9). This result indicates that tHCA is biocompatible and eligible for fluorogenic imaging in vivo given the short blood circulation half‐time of small molecules and the instantaneous reaction between TZ@SWCNTs and tHCA. Guided by the aforementioned results, we investigated the fluorogenic performance of tHCA in live cells. MCF‐7 cells were preincubated with 50 μm TZ@SWCNTs for 6 hours prior to tHCA treatment. Accumulation of TZ@SWCNTs inside cells was visualized by labelling TZ@SWCNTs with Cy3 fluorophore for confocal fluorescence imaging (Figure S10). Given the advantage of low background from tHCA and the instantaneous cleavage reaction between tHCA and TZ@SWCNTs, we carried out real‐time fluorescence imaging immediately after exposure of cells to PBS containing 1 μm tHCA. MCF‐7 cells treated sequentially with TZ@SWCNTs and tHCA showed a notable fluorescence signal in 5 minutes (Figure S11) post tHCA addition, and the fluorescence intensity increased over 30 minutes (Figure 5 a), whereas cells treated with tHCA alone showed negligible fluorescence (Figure 5 b). After 30 minutes of tHCA treatment, pretargeted cells induced a significant fluorescence enhancement by a factor of 6.5 relative to cells treated with tHCA alone (Figure 5 d). Furthermore, considering the liberated HCA is positively charged and prone to accumulate in mitochondria, we investigated the intracellular localization of liberated HCA using mitochondrial probe MitoSpy™ Green (Figure 5 c). MitoSpy™ Green is a green‐fluorescing stain that localizes to mitochondria regardless of mitochondrial membrane potential. As shown in Figure 5 e, the NIR fluorescence signal from liberated HCA colocalized well with the MitoSpy™ Green, with a Pearson's correlation coefficient of 0.92. These results indicate the efficient and fast cell permeability of tHCA probe and the almost instantaneous fluorescence signal in cells containing TZ@SWCNTs with no need for washing. The mitochondria‐targeted property of the turn‐on tHCA probe brings further potential in mitochondria‐based applications.

### Pretargeted fluorogenic in vivo NIR tumour imaging

**Figure 6:**
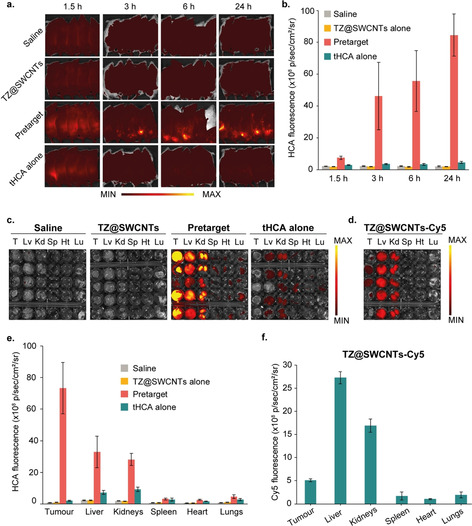
In vivo fluorescence imaging of tHCA via pretargeted strategy in mice. Mice bearing subcutaneous CT26 xenografts on the right flank (*n*=5). a) Non‐invasive fluorescence images of tumour‐bearing mice over time and b) time‐dependent tumour‐specific NIR fluorescence intensity in live mice treated with saline, TZ@SWCNTs alone, pretargeted strategy, or tHCA alone at different time points post‐tHCA injection (*n*=5). c) Ex vivo fluorescence images of dissected tumours and major organs (liver, lungs, spleen, kidneys, heart) and e) release profiling of tHCA 24 h after tHCA injection. d) Ex vivo fluorescence images of dissected tumours and major organs and f) the biodistribution of Cy5 from mice treated with TZ@SWCNTs‐Cy5. Fluorescence is expressed in radiance: photons/second/cm^2^/steradian.

Based on the successful NIR fluorogenic imaging in live cells with our two‐component strategy, we performed tumour‐pretargeted turn‐on fluorescence imaging in living mice with tumour xenografts. CT26 tumour (undifferentiated colon carcinoma cell line)‐bearing BALB/c mice were randomly allocated into 5 groups (*n*=5) when the tumour volume reached approximately 300 mm^3^. The pretargeted imaging group received a dose of 25 μmol kg^−1^ TZ@SWCNTs and a dose of 2.4 μmol kg^−1^ tHCA after a 2 hour interval, both through intravenous injection. The time lag of 2 hours was applied to allow TZ@SWCNTs to be cleared from the blood circulation and accumulate in the tumour regions via the EPR effect. TZ@SWCNTs alone, tHCA alone, and saline were also injected in parallel as control groups. In vivo fluorescence images of live mice were obtained at various time points after tHCA injection using an IVIS Lumina fluorescence/bioluminescence imaging system. For mice treated with the pretargeted imaging strategy (TZ@SWCNTs tumour‐pretargeting and subsequent tHCA injection), an indistinct signal was observed in the abdomen at 1.5 hours and became negligible at 3 hours post tHCA injection (Figure 6 a). In tumour regions, pronounced NIR fluorescence signals emerged at 3 hours post tHCA injection, and continued to increase over the course of 24 hours, indicating the release of HCA in the tetrazine‐tagged tumours. The fluorescence quantification of the tumours gave a tumour‐to‐background ratio of 23:1 at 3 hours post tHCA injection and the ratio increased to 42:1 after 24 hours (Figure 6 b). In contrast, for mice treated with tHCA alone, only very low emission levels in mice abdomen were seen 1.5 hours post tHCA injection and the signal was negligible after 3 hours. This result indicated rapid clearance of tHCA probe from the body and its specific activation in tetrazine‐tagged tumours. Notably, a much lower dose of tetrazine is required by this strategy compared to the utilization of small molecule tetrazines and even antibody‐tagged tetrazines3c, 5 because of the high tumour delivery efficiency of TZ@SWCNTs. Furthermore, the result showed that an interval of 2 hours between the administration of the two components is sufficient due to fast clearance of SWCNTs from the blood circulation,^9^ which is much shorter than the 48 hours required by antibody‐facilitated tetrazine/TCO cleavage‐reaction‐triggered activation.^5^

After 24 hours post tHCA injection, the mice were euthanized, and the tumours and major organs were harvested for ex vivo fluorescence measurements and assessment. The fluorescence images of the tumours and major organs were obtained to evaluate the in vivo release profile of tHCA as shown in Figures 6 c and 6 e. The pretargeted strategy induced a remarkable fluorescence signal in the tetrazine‐tagged tumours. The other organs, such as liver and kidneys, showed much weaker fluorescence signals. Notably, the signal in the tumours was 3.6 times higher than that in liver. These non‐specific signals were presumed to be from the reaction between tHCA and TZ@SWCNTs residues retained in the liver and kidneys during renal and hepatic clearance from the body. On the other hand, for mice treated with tHCA alone, we only observed negligible signals in the tumours and weak signals in the liver and kidneys. This result further confirmed that the activation of the fluorescent signal is tetrazine‐dependent and thus tumour specific.

To compare the pretargeted activation strategy with direct targeted delivery, a group of mice were treated with a single dose of TZ@SWCNTs‐Cy5 at a TZ dose of 25 μmol kg^−1^ and a corresponding Cy5 dose of 0.7 μmol kg^−1^ for parallel study. The ex vivo fluorescence image of tumours and major organs at 26 hours post TZ@SWCNTs‐Cy5 injection are shown in Figure 6 d, and the corresponding fluorescence intensities are shown in Figure 6 f. The fluorescence signals of TZ@SWCNTs‐Cy5 in the liver and kidneys were higher than in the tumours. The resulting tumour‐to‐liver ratio is 0.18, which is about 20 times lower than that of the pretargeted activation strategy. This result demonstrated that, compared to the direct tumour‐targeted fluorophore delivery, the two‐step activation strategy largely avoided the signals of the active probe in normal tissues and enabled greater tumour specificity.

## Conclusion

Our work presents a new pretargeted activation strategy that integrates SWCNTs and an IEDDA click‐to‐release reaction to achieve tumour‐specific delivery of active imaging agents and anticancer drugs with spatiotemporal control. Furthermore, we described the use of a bioorthogonally applicable fluorogenic NIR probe for real‐time in vivo imaging in which fluorescence was turned‐on in response to tetrazines. In this pretargeted activation strategy, the TZ@SWCNTs accumulate specifically in tumour cells as a result of the EPR effect and tagged the tumour region with the bioorthogonal tetrazine handle. The tetrazine handle serves as the trigger to react in situ with the TCO conjugate to release the active effector molecules. TZ@SWCNTs displayed an exceptionally high loading efficiency, accelerated activation rate, and improved biocompatibility relative to the corresponding small molecule mTZ. As shown in this study, our pretargeted activation strategy enabled selective tDOX activation in cells chemically tagged with tetrazines. Taking advantage of the pretargeted activation strategy and our tHCA fluorogenic probe, we have developed a NIR fluorescence imaging technique in living cells with an instantaneous turn‐on signal in mitochondria with no need for washing. With sequential systemic administration of TZ@SWCNTs and tHCA in living mice with xenograft tumours, the tetrazine‐triggered fluorogenic imaging resulted in higher tumour selectivity over liver and kidneys compared to direct fluorophore delivery. The distinct fluorescent signals in tumour regions lasted up to 24 hours after intravenous administration of the probe. Our results demonstrate that activatable NIR imaging guarantees tumour selectivity over normal tissues and facilitates in‐depth imaging with a high signal‐to‐noise ratio in a real‐time and non‐destructive manner. We anticipate that the tumour‐targeting efficiency would be potentially improved further by incorporating tumour‐targeting moieties. Moreover, with its fluorogenic property in instantaneous response to tetrazines and its mitochondria‐oriented nature, tHCA has the potential to be used for other diagnostic and imaging applications, such as fluorescence‐guided tumour surgery, super‐resolution bioimaging and high‐throughput screening, as well as in combination with other tetrazine‐containing platforms such as antibodies. Finally, we expect that this strategy could be applied to activate other TCO‐caged effector molecules for both diagnostic and therapeutic applications.

## Conflict of interest

The authors declare no conflict of interest.

## Supporting information

As a service to our authors and readers, this journal provides supporting information supplied by the authors. Such materials are peer reviewed and may be re‐organized for online delivery, but are not copy‐edited or typeset. Technical support issues arising from supporting information (other than missing files) should be addressed to the authors.

Supplementary
