# Supplementary material for: Tetrazine Carbon Nanotubes for Pretargeted In Vivo “Click‐to‐Release” Bioorthogonal Tumour Imaging
Source: Angew Chem Int Ed Engl. 2020 Aug 7;59(37):16023–32. doi: 10.1002/anie.202008012 (PMC7540421; doi:10.1002/anie.202008012)
Supplement: Supplementary file 1 — Supplementary [file ANIE-59-16023-s001.pdf]

Angewandte Chemie
Eine Zeitschrift der Gesellschaft Deutscher Chemiker

Supporting Information

Tetrazine Carbon Nanotubes for Pretargeted In Vivo "Click-to-Release" Bioorthogonal Tumour Imaging

*He Li, João Conde, Ana Guerreiro, and Gonçalo J. L. Bernardes**

anie_202008012_sm_miscellaneous_information.pdf

Supporting Information

Table of Contents

1. General Experimental Section S3

2. Procedure for TZ@SWCNTs conjugation S4

3. Synthesis and characterization of compounds.. S6

4. Restoration of fluorescence and kinetics measurements................................ S11

5. Stability and reactivity of tetrazines and tHCA S15

6. Pretargeted release in vitro S17

7. Pretargeted fluorogenic imaging in vivo S23

8. NMR spectra S25

9. References... S32

1. General Experimental Section

HiPco™ purified, small diameter SWNTs was purchased from NanoIntegris. Methyl tetrazine amine, HCOOH-salt was purchased from Sirius Fine Chemical (Cat. No. SC-1191). 3-(N-succinimidyloxyglutaryl) aminopropyl, polyethyleneglycol-carbamyl distearoylphosphatidyl-ethanolamine (DSPE-PEG2000-NHS) was purchased from NOF Europe GmbH (Cat. No. SUNBRIGHT®DSPE-020GS). 2-ax-*trans*cyclooctenol (axial (*E*)-Cyclooct-2-enol) was purchased from Sirius Fine Chemicals (Cat. No. SC-8013). All the other reagents were purchased from either Sigma–Aldrich or Fluorochem and used without further purification. Analytical thin layer chromatography was performed using Merck silica gel 60 F254 coated glass or aluminum plates and visualized by UV lamp (λ_{max} = 254 nm) and/or ninhydrin stain and potassium manganite stain as appropriate. Flash column chromatography was performed using silica gel 60 Å (mesh 230 – 400) from Material Harvest.

^{1}H NMR, ^{13}C NMR spectra were taken on a Bruker 400 MHz DPX–400 Dual Spectrometer and Bruker 500 MHz AVIII HD Smart Probe in the stated solvents. Chemical shifts are reported in parts per million (ppm) and the spectra are calibrated to the residual solvent peak ^{1}H NMR: $CDCl_3$ δ 7.26 ppm; ^{13}C NMR: $CDCl_3$ δ 77.16 ppm. Multiplicities are described as s (singlet), d (doublet), t (triplet), q (quartet), m (multiplet), dd (double doublet) and so on. Coupling constants (J) are reported in hertz (Hz) to 1 decimal place using MestReNova for signal processing. The center of each peak is reported except for multiplet signals where a range of ppm values is given. Structural assignments are made with the aid of COSY, HSQC, and HMBC experiments, performed by the NMR Spectrometry Service, University of Cambridge. High-resolution mass spectra were performed by the Mass Spectrometry Service, Department of Chemistry, University of Cambridge using a Waters LCT Premier or a Waters Xevo G2-S spectrometer and ionized by ESI. The HPLC/UV analysis was performed on a Dionex Ultimate 3000 system with a μBondapak C18 column (150 x 3.9 mm, 125Å, 10 μm, Waters). The HPLC-PDA/MS analyses were performed on a Waters Acquity UPLC system (Acquity UPLC BEH C18 column, 1.7 μm, 2.1 mm × 50 mm) coupled with a photodiode array detector and a Xeno G2-S

TOF mass spectrometer. Fluorescence spectra were obtained by Cary Eclipse Fluorescence Spectrophotometer with a xenon flash lamp and 1.0-cm quartz cells. Absorption spectra were measured on Varian Cary 400 Scan UV–Visible Spectrophotometer. The fluorescence intensity of coumarin and HCA was measured on a SpectraMax i3x (Molecular Devices) microplate reader. The fluorescent images were captured on a Leica SP5 confocal microscope.

2. Procedure for TZ@SWCNTs conjugation

2.1 Synthesis of DSPE-PEG-Tetrazine

Methyl tetrazine amine, HCOOH-salt (41 mg, 0.166 mmol, 5.0 equiv.) was dissolved in 5 mL CH_2Cl_2/DMF/MeOH (75/12/12). DSPE-PEG2000-NHS (100 mg, 0.033 mmol, 1.0 equiv.) and triethylamine (232 μL, 50 equiv.) was added. The reaction was stirred overnight at room temperature. The volatile material was removed by evaporation *in vacuo*. Water (10 mL) was added to the reaction mixture. The solution was centrifuged to remove trace insoluble. The supernatant was dialyzed in Slide-A-Lyzer^{TM} dialysis cassettes (molecular weight cutoff of 3,000 Da) (ThermoFisher) against water (5 × 2000 mL). The dialyzate was lyophilized to give pink solid. **^{1}H NMR** (500 MHz, $CDCl_3$) δ 8.57 – 8.51 (d, J = 8.4 Hz, 2H), 7.51 (d, J = 8.5 Hz, 2H), 6.76 (s, 1H), 6.52 (s, 3H), 5.19 (s, 1H), 4.55 (d, J = 5.9 Hz, 2H), 4.36 (d, J = 9.5 Hz, 2H), 4.14 (dd, J = 11.9, 6.9 Hz, 4H), 3.97 (s, 8H), 3.75 – 3.59 (m, 195H), 3.60 – 3.53 (m, 8H), 3.49 (dd, J = 8.8, 4.2 Hz, 3H), 3.42 – 3.31 (m, 5H), 3.09 (s, 3H), 2.40 – 2.32 (m, 4H), 2.32 – 2.22 (m, 13H), 1.99 (p, J = 7.2 Hz, 4H), 1.77 (dt, J = 11.6, 5.8 Hz, 4H), 1.63 – 1.51 (m, 11H), 1.25 (s, 60H), 0.88 (t, J = 7.0 Hz, 6H) ppm. **^{13}C NMR** (126 MHz, $CDCl_3$) δ 245.58, 173.50, 173.10, 172.93, 172.67, 167.36, 164.03, 143.76, 130.92, 128.61, 128.33, 70.71, 70.59, 70.36, 70.20, 43.28, 38.27, 35.62, 35.42, 34.39, 34.22, 32.08, 29.87, 29.82, 29.72, 29.52, 29.34, 28.95, 25.09, 25.03, 22.84, 22.19, 21.31, 14.28 ppm.

2.2 Functionalization of SWCNTs with DSPE-PEG-Tetrazine

Pristine HiPco SWCNTs (0.2 mg/mL) were sonicated in an aqueous solution of DSPE-PEG2000-NHS (0.2 mg/mL) and DSPE-PEG-TZ (2 mg/mL) using an 80 kHz bath sonicator (Elmasonic P60H Ultrasonic Bath) for 1 h with the bath

temperature kept below 20 ºC. The solution was centrifuged at 16,000 g for 20 min. The supernatant solution was collected to give SWCNT-DSPE-PEG-TZ complexes (TZ@SWCNTs) and stored at 4 ºC. TZ@SWCNTs was washed with Milli-Q water three times using centrifugal filter (Amicon Ultra-4, 100 KDa MWCO) to remove the excess DSPE-PEG-TZ before use. As a control, SWCNTs (0.2 mg/ml) was sonicated with DSPE-PEG-NHS (2 mg/mL) to give SWCNT-DSPE-PEG-COOH complexes (SWCNTs).

2.3 Quantification of Tetrazines on SWCNTs

The tetrazine loading efficiency was determined by quantitative UV spectrophotometry of the DSPE-PEG-TZ in water. Standard curve for absorption of DSPE-PEG-TZ at 270 nm with concentration from 0 to 150 mg/L was measured, giving an extinction coefficient of 0.0946 mg^{-1}*L*cm^{-1} (equals 292.8 $mmol^{-1}$*L*cm^{-1}). After sonicating DSPE-PEG-TZ (2 mg/mL) and DSPE-PEG-NHS (0.2 mg/mL) with SWCNTs (0.2 mg/mL), the unattached DSPE-PEG-TZ was collected by filtration through a centrifugal filter (Amicon Ultra-4, 100 KDa MWCO) . The concentration of DSPE-PEG-TZ in the filtration was determined by the absorbance at 270 nm and fitting into the standard curve. The concentrations of SWCNTs were determined by the absorbance at 808 nm with an extinction coefficient of 0.0465 mg^{-1} L cm^{-1}.[1]

2.4 Transmission electron microscopy (TEM) characterization of TZ@SWCNTs

TZ@SWCNTs solution of SWCNTs concentration of 20 mg/L was dropped on top of a TEM grid several times, dried in the air and imaged by Thermo Scientific (FEI) Talos F200X G2 transmission electron microscope.

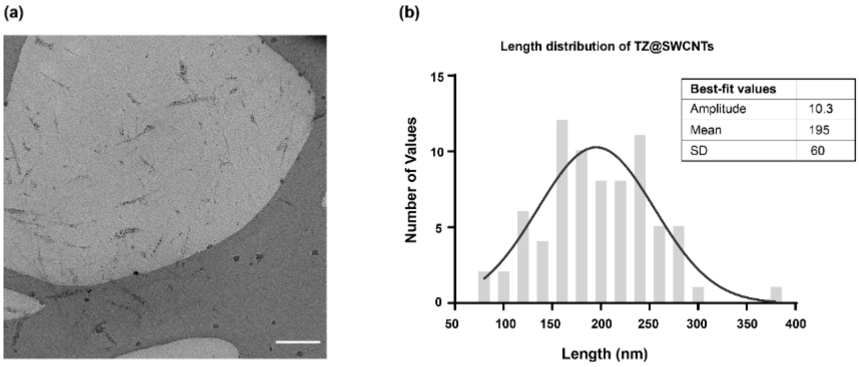

Figure S1. (a) TEM image of TZ@SWCNTs by directly dispersing TZ@SWCNTs in water. Scale bar: 200 nm. (b) Histogram plot shows the length distribution of TZ@SWCNTs in the TEM images.

3. Synthesis and characterization of compounds

3.1 Synthesis of tAMC

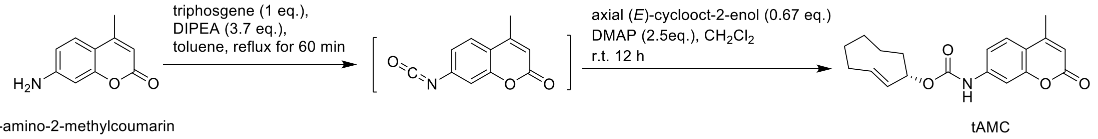

tAMC was synthesized according to the reference[2] with some modifications. 7-amino-2-methylcoumarin (64.3 mg, 0.367 mmol, 1.0 equiv.) was dissolved in toluene (12 mL), followed by adding triphosgene (108.9 mg, 0.367 mmol, 1.0 equiv.) and N, N-diisopropylethylamine (175.5 mg, 1.36 mmol, 3.7 equiv.). The mixture was refluxed at 120 °C for 60 min to get 7-isocyanate-2-methylcoumarin. The volatile material was removed by evaporation *in vacuo*.

Axial (*E*)-cyclooct-2-enol (31.0 mg, 0.246 mmol, 0.67 equiv.) was dissolved in anhydrous CH_2Cl_2 (10 mL), followed by adding 4-(N, N-dimethylamino) pyridine (112.1 mg, 0.917 mmol, 2.5 equiv.) under ice-water bath. To this mixture, 7-isocyanate-2-methylcoumarin from last step was added. The reaction was stirred at room temperature in the dark for 12 h. Then the mixture was diluted with 50 ml CH_2Cl_2 and washed with H_2O (x4), dried over anhydrous Na_2SO_4 and concentrated *in vacuo*. The product was then purified by silica chromatography (MeOH/CH_2Cl_2=1%) to give tAMC (40 mg, 0.12 mmol, 50%) as a white solid. **^{1}H NMR** (400 MHz, $CDCl_3$) δ 7.53 (d, J = 8.7 Hz, 1H), 7.48 – 7.36 (m, 2H), 6.18 (q, J = 1.3 Hz, 1H), 5.89 (ddd, J = 15.6, 11.1, 3.8 Hz, 1H), 5.58 (dd, J = 16.4, 2.4 Hz, 1H), 5.47 (s, 1H), 2.49 (d, J = 10.4 Hz, 1H), 2.41 (d, J = 1.3 Hz, 3H), 2.15 (dd, J = 15.3, 4.9 Hz, 1H), 2.03 (td, J = 14.2, 12.4, 6.8 Hz, 2H), 1.97 – 1.63 (m, 4H), 1.56 – 1.43 (m, 1H), 1.12 (td, J = 14.1, 6.0 Hz, 1H) ppm; **^{13}C NMR** (126 MHz, $CDCl_3$) δ 161.24, 154.60, 152.35, 152.30, 141.65, 132.51, 130.72, 125.53, 115.61, 114.45, 113.28, 105.93, 75.18, 40.78, 36.15, 36.09, 29.18, 24.35, 18.73 ppm. **HRMS** (ESI$^+$) calcd for $C_{19}H_{22}NO_4$ $[M+H]^+$ 328.1549, found 328.1546.

3.2 Synthesis of tDOX

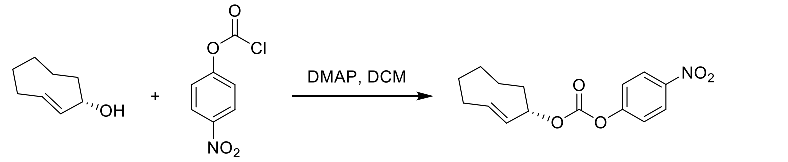

tDOX was synthesized according to the reference.[3] A solution of axial (*E*)-cyclooct-2-enol (16 mg, 0.127 mmol, 1.0 equiv.) in 1 mL CH_2Cl_2 was cooled on ice. 4-(N, N-dimethylamino) pyridine (38.7 mg, 0.317 mmol, 2.5 equiv.) was added under argon protection, followed by 4-nitrophenylchloroformate (51 mg, 0.254 mmol, 2.0 equiv.). The solution was stirred overnight. A saturated solution of NH_4Cl (2.5 mL) was used to stop the reaction. After phase separation, the aqueous layer was extracted with CH_2Cl_2. The combined organic layers were washed with a saturated aqueous NaCl solution, dried over $NaSO_4$, and concentrated *in vacuo*. The crude product was blended with 300 mg silica gel

in 1 ml CH_2Cl_2, gently evaporated *in vacuo* and then subjected to silica chromatography (0 – 5% EtOAc/petrol ether) to yield (*E*)-cyclooct-2-en-1-yl (4-nitrophenyl) carbonate as an oil (28.5 mg, 0.096 mmol, 75 %). **^{1}H NMR** (500 MHz, $CDCl_3$): δ 8.28 (m, 2H), 7.40 (m, 2H), 5.98 (m, 1H), 5.56 (dd, 1H), 5.44 (br, 1H), 2.45 (m, 1H), 2.53 (m, 1H), 2.21 (m, 1H), 2.13 –1.86 (m, 3 H), 1.85 – 1.50 (m, 2H), 0.95 – 0.79 (m, 2 H) ppm. **^{13}C NMR** (126 MHz, $CDCl_3$): δ 155.62, 151.64, 145.30, 133.15, 129.34, 125.28, 121.80, 78.79, 40.46, 35.98, 28.93, 23.97 ppm.

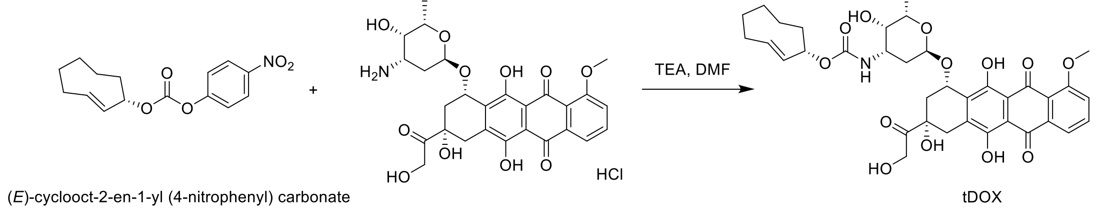

The (*E*)-cyclooct-2-en-1-yl (4-nitrophenyl) carbonate (6 mg, 0.0167 mmol, 1.0 equiv.) was dissolved in DMF (3 ml). Doxorubicin hydrochloride (10.9 mg, 0.0189 mmol, 1.13 equiv.) and triethylamine (25.6 μL, 0.184 mmol, 11 equiv.) were added. The reaction mixture was stirred in the dark at room temperature under argon atmosphere for 18 h. The volatile material was removed by evaporation *in vacuo.* The residue was dissolved in CH_2Cl_2washed with saturated $NaCO_3$, distilled water (2×) and saturated NaCl solution (2×). The organic layer was dried over $NaSO_4$, concentrated *in vacuo* and purified by silica chromatography (0 – 5% MeOH/CH_2Cl_2) to give tDOX as pink powder (8.3 mg, 0.0119 mmol, 60%). **^{1}H NMR** (500 MHz, $CDCl_3$) δ 13.97(s, 1H), 13.23(s, 1H), 8.02(d, 1H), 7.78(dd, 1H), 7.39(d, 1H), 5.75(m, 1H), 5.51(s, 1H), 5.47(m, 1H), 5.29(d, 1H), 5.24(s, 1H), 5.09 (br, 1H), 4.75(s, 2H), 4.53(s, 1H), 4.14(q, 1H), 4.08 (s, 3H), 3.87 (m, 1H), 3.68 (s,1H), 3.25(d, 1H), 3.02(s, 1H), 3.00(d, 1H), 2.43(m, 1H), 2.32(d, 1H), 2.17(d, 1H), 2.1-1.7 (m, 6H), 1.61(m, 3H), 1.45(m, 1H), 1.30(d, 3H), 1.08-0.96(m, 1H), 0.75 (m, 1H) ppm. **^{13}C NMR** (126 MHz, $CDCl_3$) δ 214.06, 187.25, 186.84, 161.20, 156.31, 155.81, 155.15, 135.91, 135.66, 133.74, 133.69, 131.91, 131.42, 121.03, 120.00, 118.58, 111.74, 111.55, 100.88, 74.25, 69.86, 69.76, 67.45, 65.70, 56.83, 46.95, 40.76, 36.02,

35.81, 34.18, 30.37, 29.85, 29.21, 24.20, 17.00 ppm. **HRMS**(ESI^+) calcd for $C_{36}H_{41}NO_{13}Na$ $[M+Na]^+$ 718.2476, found 718.2491.

3.3 Synthesis of HCA

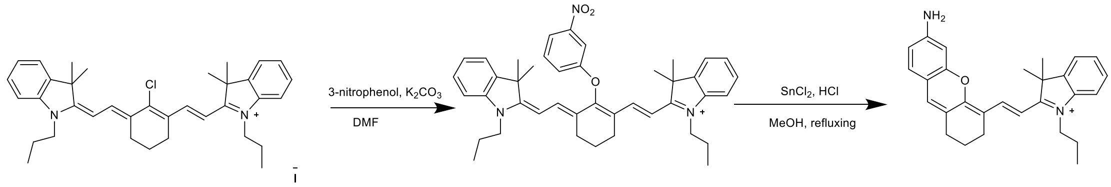

IR780 IR780-NP HCA

Hemicyanine (HCA) was synthesized according to the reference[4] with some modifications. 3-Nitrophenol (104.2 mg, 0.75 mmol, 5.0 equiv.) and K_2CO_3 (103.6 mg, 0.75 mmol, 5.0 equiv.) were dissolved in 2 mL DMF in a flask, and the mixture was stirred at room temperature under argon atmosphere for 10 min. Then, the mixture was added to IR-780 iodide (100 mg, 0.150 mmol, 1.0 equiv.) in DMF (4 mL) via a syringe and the mixture was stirred at room temperature for another 12 h under argon atmosphere. The reaction mixture was extracted with CH_2Cl_2 and washed with 0.2 M HCl (3×), distilled water (2×) and saturated NaCl solution (2×). The organic layer was dried over $NaSO_4$, concentrated *in vacuo* and purified by silica chromatography (0 – 6% MeOH/ CH_2Cl_2) to give **IR780-NP** as a green powder (86 mg, 0.112 mmol, 75%). **^{1}H NMR** (500 MHz, $CDCl_3$) δ 7.97(t, 1H), 7.92 (dd, 1H), 7.78 (d, 2H), 7.62 (t, 1H), 7.44 (dd, 1H), 7.35 (m, 2H), 7.25 (s, 2H), 7.19 (t, 2H), 7.11 (d, 2H), 6.26(d, 2H), 4.16 (t, 4H), 2.81 (t, 4H), 2.08(t, 2H), 1.87(m, 4H), 1.34(s, 12H), 1.05 (t, 6H) ppm. **^{13}C NMR** (126 MHz, CDCl3) δ 172.10, 161.93, 159.86, 149.59, 142.42, 141.07, 140.98, 131.73, 128.87, 125.33, 122.40, 122.25, 121.20, 117.53, 110.99, 110.29, 101.32, 49.11, 46.32, 28.04, 24.71, 21.21, 21.07, 11.78 ppm.

$SnCl_2$ (152.8 mg, 0.8 mmol, 20 equiv.) was dissolved in concentrated HCl (0.163 mL, 2.0 mmol, 50 equiv.), followed by adding compound **IR780-NP** (30 mg, 0.04 mmol, 1.0 equiv) in MeOH (6 ml) under argon atmosphere. The reaction solution was refluxed at 70 ºC under argon atmosphere overnight. The volatile material was removed by evaoparation *in vacuo*. The residue was extracted with CH_2Cl_2 and washed with 0.2 M HCl (3×), distilled water (2×) and saturated NaCl solution (2×). The organic layer was dried over $NaSO_4$,

concentrated *in vacuo* and purified by silica chromatography (0 – 8% MeOH/CH_2Cl_2) to give HCA as a green solid (19 mg, 90%). **^{1}H NMR** (500 MHz, $CDCl_3$) 8.49(d, 1H), 7.41 (d, 1H), 7.39 (s, 1H), 7.36 (m, 1H), 7.24-7.21 (m, 2H), 7.07 (s, 1H), 7.05 (d, 1H), 6.96 (d, 1H), 5.94 (d, 1H), 3.98 (t, 2H), 2.74(t, 2H), 2.63(t, 2H), 1.97-1.85(m, 4H), 1.76 (s,6H), 1.06 (t, 3H) ppm. **^{13}C NMR** (126 MHz, $CDCl_3$) 172.98, 164.01, 156.74, 156.03, 142.34, 141.68, 141,06, 140.10, 129.57, 128.65, 125.25, 122.84, 122.02, 116.68, 114.64, 114.20, 110.22, 99.10, 98.18, 49.61, 45.86, 28.95, 28.73, 24.44, 20.89, 20.78, 11.84ppm. **HRMS**(ESI$^+$) calcd for $C_{28}H_{31}N_2O^+$ [M]$^+$: 411.2437, found: 411.2436.

3.4 Synthesis of tHCA

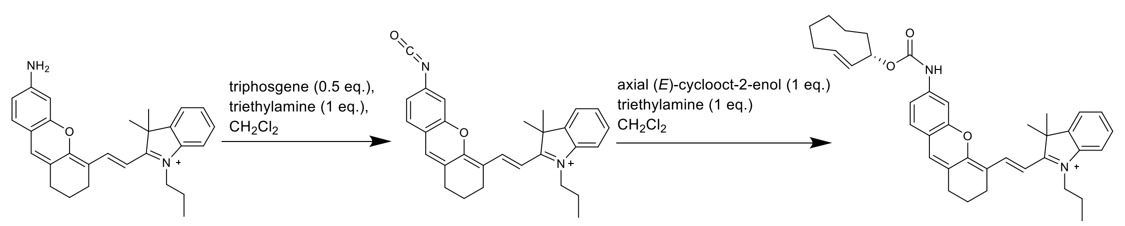

Triphosgene (14.4 mg, 0.049 mmol, 0.5 equiv.) was dissolved in 1 mL anhydrous $CH_2Cl_2$and cooled on ice, followed by adding dropwise HCA (40 mg, 0.097 mmol, 1.0 equiv.) in anhydrous CH_2Cl_2 (3 mL) and triethylamine (0.097 mmol, 0.014 mL, 1.0 equiv.) under N_2 protection. The mixture was warmed to 25 ºC and stirred for another 3 h under N_2 protection. The volatile material was evaporated *in vacuo* and put under high vacuum for another 30 min. The crude was used immediately for the next step.

Axial (*E*)-cyclooct-2-enol (13.2 mg, 0.01 mmol, 1.0 equiv.) was dissolved in anhydrous CH_2Cl_2 (0.5 mL), followed by adding product from last step (in 4 mL CH_2Cl_2) and triethylamine (0.014 mL, 0.097 mmol, 1.0 equiv.,) in 1 mL CH_2Cl_2on ice under N_2 protection. The reaction was stirred at room temperature in the dark overnight. Then the reaction mixture was extracted with $CH_2Cl_2$and washed with water for 4 times. The organic layer was dried over $NaSO_4$, concentrated *in vacuo* and purified by silica chromatography (0 – 8% MeOH/CH_2Cl_2) to give tHCA as a cyan powder (8.4 mg, 0.014 mmol, 14%). **^{1}H**

NMR (500 MHz, $CDCl_3$) δ 8.61 (d, J = 11.0 Hz, 1H), 8.18 (s, 1H), 7.98 (d, J = 8.7 Hz, 1H), 7.48 (d, J = 7.1 Hz, 1H), 7.43 (m, 1H), 7.35 (m, 1H), 7.29 (d, J = 8.6 Hz, 1H), 7.24 (m, 2H), 6.31 (d, J = 10.8 Hz, 1H), 6.20 (ddd, J = 15.7, 11.1, 3.8 Hz, 1H), 5.50 (dd, J = 16.4, 2.3 Hz, 1H), 5.31 (s, 1H), 4.42 – 4.18 (m, 2H), 2.71 (m, 2H), 2.65 (m, 2H), 2.49 (m, 1H), 2.26 – 2.09 (m, 1H), 2.07 – 1.84 (m, 6H), 1.80 (d, J = 7.5 Hz, 6H), 1.77 – 1.31 (m, 6H), 1.08 (t, J = 7.4 Hz, 3H). **^{13}C NMR** (126 MHz, $CDCl_3$) δ 176.71, 162.86, 154.20, 153.77, 145.43, 141.80, 135.76, 133.48, 130.26, 128.99, 127.95, 126.83, 126.81, 122.82, 118.29, 114.79, 116.65, 111.95, 105.09, 102.35, 74.71, 50.58, 46.89, 40.91, 36.31, 35.79, 29.43, 29.20, 28.74, 24.39, 24.04, 21.28, 20.55, 11.80. **HRMS**(ESI^+) calcd for $C_{37}H_{43}O_3N_2^+$ $[M]^+$: 563.3268, found: 563.3256.

4. Restoration of fluorescence and kinetics measurements

4.1 Fluorescence recovery of tAMC with various tetrazines

TZ@SWCNTs was washed with Milli-Q water three times using centrifugal filter (Amicon Ultra-4, 100 KDa cutoff) to remove the excess DSPE-PEG-TZ before use.

Stock solutions: DSPE-PEG-TZ (20 mM in DMSO), mTZ (20 mM in DMSO), 7-amino-2-methylcoumarin (13.8 mM in DMSO), tAMC (13.8 mM in DMSO).

The TZ@SWCNTs, DSPE-PEG-TZ or mTZ were mixed with tAMC (20 μM) with a final tetrazine concentration of 50 μM in 10% DMSO/PBS or pure human plasma. The reactions were conducted at 37 ºC and the fluorescence intensity (F_t) was measured by SpectraMax i3x (Molecular Devices) microplate reader at given time points (ex. 380 nm, em. 445 nm). At the same time, the fluorescence (F_0) of 20 μM tAMC and the fluorescence (F_{100}) of 20 μM 7-amino-2-methylcoumarin were also measured as control experiments. The relative fluorescence (%) was calculated as $F_t/F_{100}\times100\%$. The experiment was performed in triplicate.

4.2 Reaction kinetics between tAMC and mTZ/DSPE-PEG-TZ

The rate constant of reaction between tAMC and mTZ or between tAMC and DSPE-PEG-TZ were determined under the second order conditions in MeCN/H_2O (1/1, v/v) at 20 ºC by following the decay of the UV absorption of the tetrazine at the 535 nm. Stock solutions of DSPE-PEG-TZ (20 mM), mTZ (20 mM) and tAMC (20 mM) were prepared in DMSO. A cuvette was filled with MeCN/H_2O (1/1, v/v) (285 μL) and equilibrated at 20 ºC. Then tetrazine was added, followed by a stoichiometric amount of tAMC, resulting identical final concentration of both reactants of 0.5 mM or 0.75 mM. The decay of the UV absorption of the tetrazine at 535 nm was followed over time with a Cary 400 UV-Vis spectrophotometer. From this absorption at 535 nm, the concentration of tetrazine was calculated using a molar absorption coefficient of ε = 430 M^{-1} cm^{-1}. The resulted values for $(1/c - 1/c_0)$ were then plotted against time and fitted to a linear equation to obtain the second order constant k from the slope.

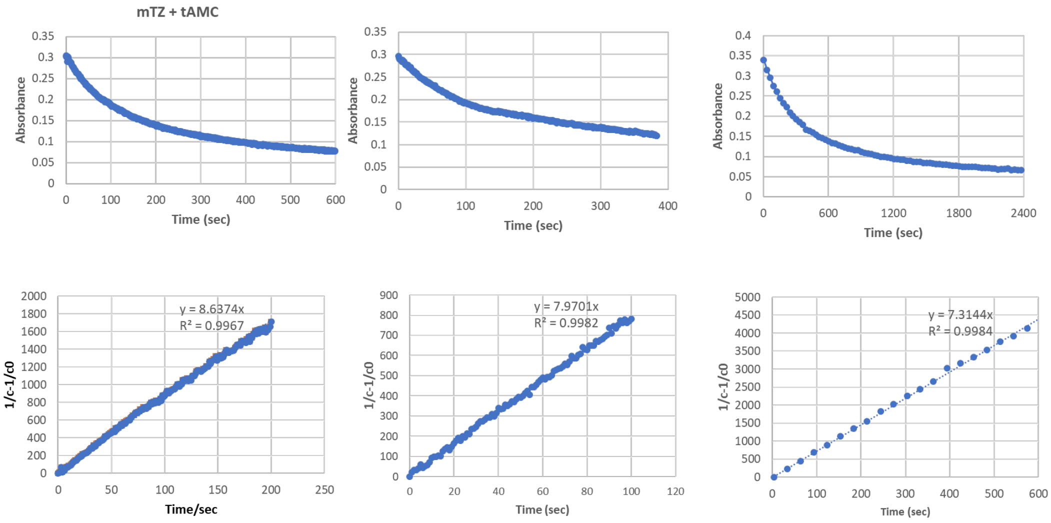

Figure S2a. Kinetic plots of reaction of mTZ with tAMC in MeCN/H_2O (1/1, v/v) at 20 ºC by UV spectroscopy at 20 ºC, c_0= 0.5 or 0.75 mM. k_2 was calculated to be 7.97 ± 0.66 M^{-1} s^{-1}.

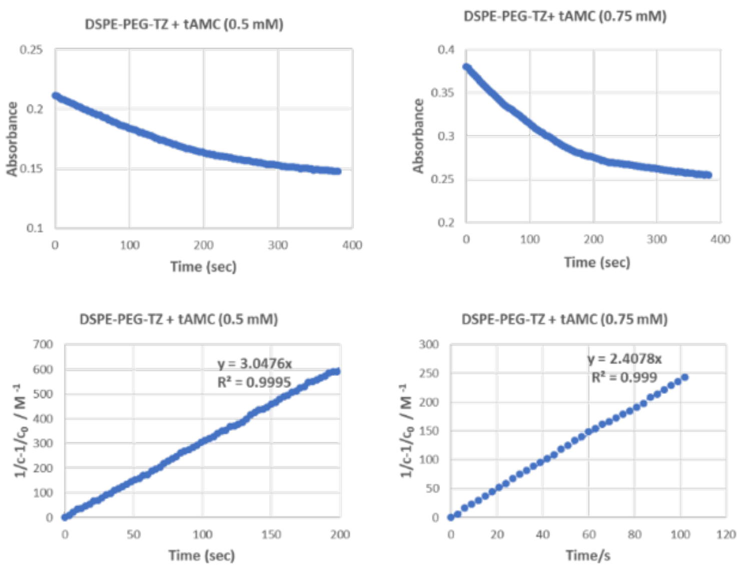

Figure S2b. Kinetic plots of reaction of DSPE-PEG-TZ with tAMC in MeCN/H_2O (1/1, v/v) at 20 ºC by UV spectroscopy at 20 ºC, c_0= 0.50 or 0. 75 mM. k_2 was calculated to be 2.73 ± 0.45 M^{-1} s^{-1}.

4.3 Absorbance and emission spectra of HCA and tHCA

Stock solutions: HCA (5.5 mM in DMSO), tHCA (6.6 mM in DMSO). The stock solution of the fluorophore was diluted to 25 μM in 10% DMSO/PBS and transferred to a quartz cell of 1 cm optical length to measure absorbance or fluorescence with λ_{ex} = 665 nm.

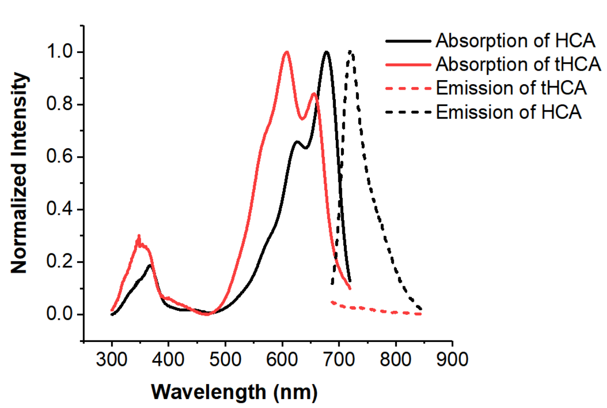

Figure S3. Absorbance and emission spectra of HCA and tHCA at concentration of 25 μM in 10% DMSO/PBS.

4.4 Fluorescence recovery of tHCA with various tetrazines

TZ@SWCNTs was washed with Milli-Q water three times using centrifugal filter (Amicon Ultra-4, 100 KDa cutoff) to remove the excess DSPE-PEG-TZ before use.

Stock solutions: DSPE-PEG-TZ (20 mM in DMSO), mTZ (20 mM in DMSO), HCA (5.5 mM in DMSO), tHCA (6.6 mM in DMSO).

The TZ@SWCNTs, DSPE-PEG-TZ or mTZ were mixed with tHCA (20 μM) with a final tetrazine concentration of 50 μM in 10% DMSO/PBS or pure human plasma. The reactions were conducted at 37 ºC and the fluorescence intensity (F_t) was measured by SpectraMax i3x (Molecular Devices) microplate reader at given time points (ex. 665 nm, em. 720 nm). At the same time, the fluorescence (F_0) of 20 μM tHCA and the fluorescence (F_{100}) of 20 μM HCA were also measured as control experiments. The relative fluorescence (%) was calculated as $F_t/F_{100}\times100\%$. The experiment was performed in triplicate.

4.5 HPLC trace of reaction between DSPE-PEG-TZ and tHCA

HPLC was performed on a Dionex Ultimate 3000 system with a μBondapak C18 column (150 x 3.9 mm, 125Å, 10 μm, Waters). The conditions were as follows: volume ratio of acetonitrile/H_2O = 20: 80 (0 min) to 80:20 (12 min); flow rate 1 mL min^{-1}; detection under UV light at 665 nm. The stock solution of probe tHCA/HCA was prepared at 5 mM in DMSO. For the reaction, tHCA was diluted with PBS (pH 7.4) to a final concentration of 50 μM, followed by addition of DSPE-PEG-TZ (final concentration 125 μM). After shaken at 37 ºC for 5 or 30 min, 100 μL reaction solution was transferred to an HPLC vial and injected onto the HPLC (inj. vol. 10 μL) and analyzed at 665 nm. For the control experiment, the compound was diluted by PBS to the relative concentration and injected onto the HPLC. All the samples were run in at least triplicate.

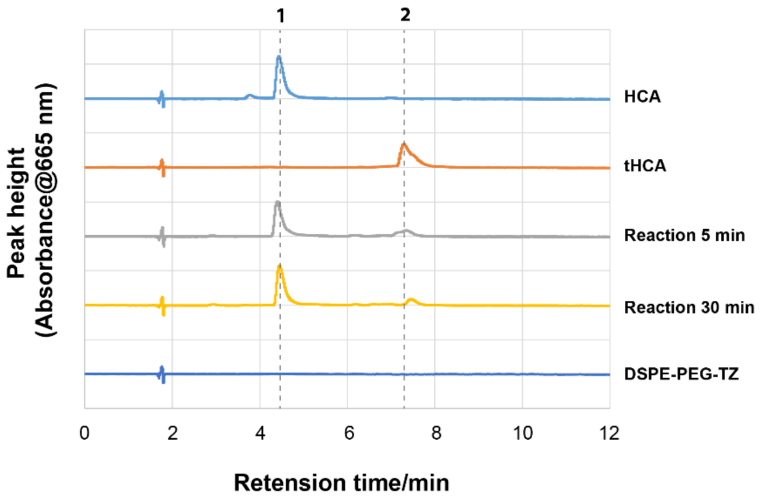

Figure S4. HPLC analysis of HCA, tHCA and the reaction of tHCA with DSPE-PEG-TZ in PBS. (Light blue) HCA (50 μM); (Orange) tHCA (50 μM); (Gray) the reaction solution of tHCA (50 μM) and DSPE-PEG-TZ (125 μM) at 37 ºC for 5 min; (Yellow) the reaction solution of tHCA (50 μM) with DSPE-PEG-TZ (125 μM) at 37 ºC for 30 min; (Dark blue) DSPE-PEG-TZ (125 μM). A volume of 10 μL was injected onto the HPLC and analyzed at 665 nm. The assignment of peaks: (1) 4.4 min, HCA; (2) 6.7 min, tHCA.

5. Stability and reactivity of tetrazines and tHCA

5.1 Stability of tetrazines in pure human plasma

Stock solutions: DSPE-PEG-TZ (20 mM in DMSO), mTZ (20 mM in DMSO), TZ@SWCNTs (20 mM in water), tHCA (6.6 mM in DMSO).

Methods: The assessments of the stability of mTZ, DSPE-PEG-TZ and TZ@SWCNTs in pure human serum were performed at 37 ºC in triplicate. A stock solution of mTZ, DSPE-PEG-TZ or TZ@SWCNTs was diluted with pure human plasma to a final concentration of 200 μM. The sample was shaken at 37 ºC for 4 h before being diluted to 20 μM with PBS (final solution is 10% human plasma/PBS). Then the stoichiometric amount of tHCA was added to the 20 μM tetrazine solutions and allowed to react at 37 ºC for 40 h before the fluorescence intensity (F_{4h}) was measured by SpectraMax i3x (Molecular

Devices) microplate reader (ex. 665 nm, em. 720 nm). The fluorescence intensity of tHCA reacted with fresh tetrazines was measure at the same time as F_{0h}. The fluorescence (F_{tHCA}) of 20 μM tHCA and the fluorescence (F_{HCA}) of 20 μM HCA were also measured as control experiments. The relative reactivity remained (%) was calculated as $(F_{4h}-F_{tHCA})/(F_{0h}-F_{tHCA}) \times 100\%$. The experiment was performed in triplicate.

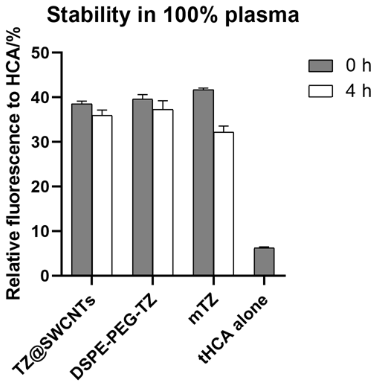

37°C, 4h incubation in 100% human plasma	Relative reactivity remained towards tHCA (%)
TZ@SWCNTs	92.0 ± 6.2
DSPE-PEG-TZ	93.1 ± 10.1
mTZ	73.2 ± 6.6

Figure S5. Stability of tetrazines in human plasma determined by reactivity remained against tHCA.

5.2 Stability of tHCA in 20% human plasma determined by HPLC.

Methods: The assessments of tHCA stability in 20% human serum/PBS were performed at 37 ºC in triplicate. A stock solution of tHCA in DMSO (6.6 mM) was diluted with 20% human plasma/PBS to a final concentration of 200 μM. The sample was shaken at 37 ºC for 0, 4, and 24 h before deproteinized by adding the same volume of cold acetonitrile. The mixture was then centrifuged at 16000 g for 10 min. The clear supernatant was analyzed by HPLC-PDA/MS to determine the intactness of tHCA. The area of peak 12.4 min was used to quantify the intactness of the probe.

General method for HPLC-PDA/MS: Water with 0.1% formic acid (solvent A) and 71% acetonitrile and 29% water with 0.075% formic acid (solvent B), were used as the mobile phase at a flow rate of 0.2 mL min^{-1}. The gradient was programmed as follows: 100% A for 2 min, from 100% A to 100% B for 9 min

then 100% B for 5 min and 100% A for 4 min. Retention time (HCA) =10.8 min; Retention time(tHCA) = 12.4 min.

Standard curve for measuring concentration of tHCA: 6.6 mM solution of tHCA was diluted in PBS to 40, 80, 120, 160 and 200 μM. The solutions were mixed with the same volume of cold acetonitrile to give final concentrations for 20, 40, 60, 80, 100 μM respectively. The solutions were centrifuged at 16000 g for 10 min and the supernatant was submitted to HPLC-PDA/MS respectively for analysis. The experiments were repeated for three times. Standard curve was made using linear regression based on the peak area ratios and concentrations.

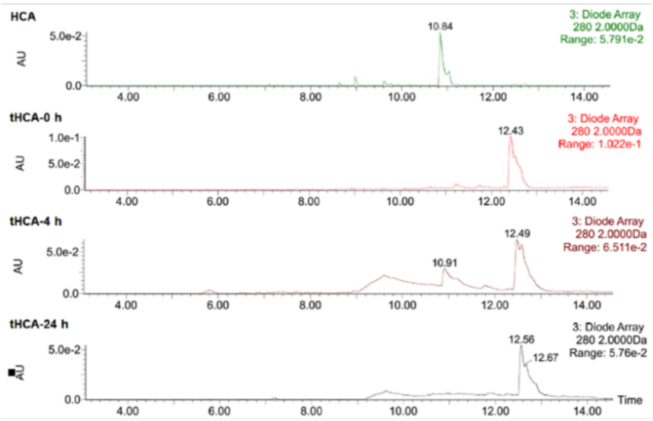

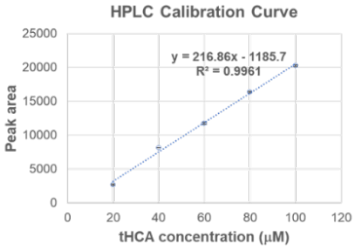

Figure. Calibration curve for tHCA. The concentration of standard tHCA solutions were plotted as a function of the corresponding peak areas.

	tHCA intact/%
4 h	81.6 ± 5.9
24h	51.6 ± 0.2

Table. Percentage of intact tHCA in 20% human plasma/PBS at 37 °C after 4h, 24h, measured by HPLC.

Figure S6. Stability of tHCA in 20% human plasma/PBS determined by HPLC-PDA/MS.

6. Pretargeted release in vitro

6.1 Cell culture

MDA-MB-231 (triple-negative breast adenocarcinoma, negative for HER/neu 2 expression), MCF-7 (breast adenocarninoma, positive for HER/neu 2 expression) and SK-BR-3 (breast adenocarninoma, HER/neu 2 over-expression) cells were grown routinely in Dulbecco's modified Eagle's medium (Cat.10569010, Gibco) supplemented with 10% heat-inactivated fetal bovine serum (Cat. A3160802, Gibco), 1x MEM non-essential amino acids (Cat.

11140035, Gibco), 100 units/mL penicillin and streptomycin in a humidified incubator at 37 ºC under 5% CO_2.

6.2 Cytotoxicity of tetrazines

MDA-MB-231, MCF-7 and SK-BR-3 cells were seeded into 96-well plates at a 5000 cells/well density 36 h prior to the experiment. The cells were treated with 1 – 200 μM mTZ, DSPE-PEG-TZ or TZ@SWCNTs for 24 h. Then the media was removed and replaced with fresh DMEM medium. The cells were incubated for another 48 h before CellTiter-Blue assay. Briefly, the medium was removed and 100 μL complete DMEM plus 20 μL CellTiter-Blue was added. Fluorescence was measured at 560ex/590em nm using a plate reader (SpectraMax i3x, Molecular Devices). The proliferation assay was performed in triplicate.

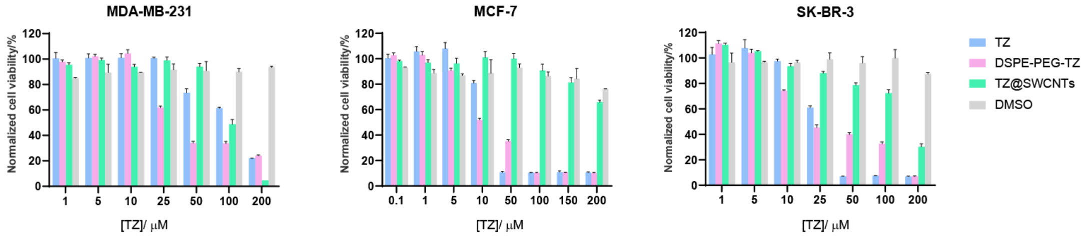

Figure S7. Cytotoxicity of mTZ, DSPE-PEG-TZ and TZ@SWCNTs. Human breast cancer cell lines MDA-MB-231, MCF-7 and SK-BR-3 were incubated with tetrazines at various concentration from 0.1 μM to 200 μM for 24 h, and the cell viability was determined by CellTiter-Blue assay (n=3; error bars represent the STD). These results showed that TZ@SWCNTs exhibits improved biocompatibility against these cells as compared to mTZ and DSPE-PEG-TZ.

6.3 Pretargeted tAMC imaging in cells

MCF-7 cells were seeded at a concentration of 30 000 cells/well in ibiTreat 8-well μ-slide (ibidi, Germany) and allowed to adhere and adapt to the slide for 36 h. At this point, culture media were changed to complete DMEM with or without 50 μM TZ@SWCNTs. After 6 h treatment, the media were removed.

Cells were washed 3 times before cultured with 200 μL of 10 μM tAMC in complete DMEM medium for another 4 h. Then cells were fixed and the nuclei were stained with 2.5 μM SYTO™ 85 orange fluorescent nucleic acid stain before imaged using Leica SP5 confocal microscope equipped with a 405 nm (coumarin) and a 561 nm (SYTO™ 85) laser units through a 63 x/1.4 HCX PL Apo CS Oil objective.

6.4 Cytotoxicity of doxorubicin (DOX), tDOX and pretargeted strategy

Cell viability was assessed using the CellTiter-Blue assay (Promega; Madison, WI). SK-BR-3, MCF-7 or MDA-MB-231 cells were seeded into 96-well plates at a 5000 cells/well density 36 h prior to the experiment. Doxorubicin (DOX) or tDOX prodrug was added at the indicated concentrations. Briefly, doxorubicin (10 mM in DMSO), tDOX (10 mM in DMSO) were diluted in pre-warmed complete DMEM immediately before the experiment and added to the wells (200 μL final volume per well). For pretargeted delivery, cells were pre-treated with 100 μL complete DMEM with TZ@SWCNTs (20 μM) for 6 h before replacing the media to 200 μL complete DMEM with different concentration of tDOX. After another 70-h period of incubation at 37 °C, the medium was removed and 100 μL complete DMEM plus 20 μL CellTiter-Blue was added. Fluorescence was measured at 560ex/590em nm using a plate reader (SpectraMax i3x, Molecular Devices). The proliferation assay was performed in triplicate. The half maximal effective concentration (EC50) values were derived from dose-response curve generated with GraphPad Prism 6.

(a)

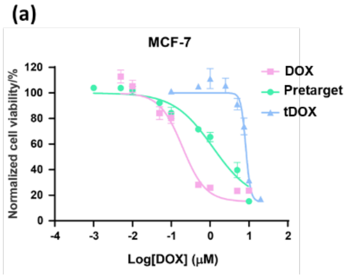

(b)

MCF-7	EC50/mM
DOX	0.190 ± 0.054
Pretarget	1.18 ± 0.31
tDOX	8.30 ± 0.42

Figure S8. (a) Cytotoxicity of doxorubicin, prodrug tDOX and pretargeted strategy on MCF-7 breast cancer cells (n=3; error bars represent the STD). Pink

and blue lines indicate cells treated with DOX or tDOX of different concentration for 72 h. For green lines, cells were pretreated with TZ@SWCNTs (20 μM) for 6 h before replacing the media to complete medium with various concentration of tDOX for another 72 h. (b) Calculated EC50 (half-maximal effective concentration) values for doxorubicin, prodrug tDOX and pretargeted strategy against MCF-7 breast cancer cells.

6.5 Cytotoxicity of TZ@SWCNTs vehicle with or without tDOX on SK-BR-3 and MDA-MB-231 cell lines

SK-BR-3 or MDA-MB-231 cells were seeded into 96-well plates at a density of 5000 cells/well 36 h prior to the experiment. The cells were treated with 1 - 100 μM TZ@SWCNTs for 6 h. Then the media was removed and replaced with fresh DMEM medium with or without 1 μM tDOX. The cells were incubated for another 70 h before CellTiter-Blue assay. The proliferation assay was performed in triplicate.

6.6 Cytotoxicity of tHCA

MCF-7 cells were seeded into 96-well plates at a density of 5000 cells/well 36 h prior to the experiment. The cells were treated with 0.5 - 5 μM tHCA in complete DMEM for 2 or 6 h. Then the media was removed and replaced with fresh DMEM medium. The cells were incubated for another 72 h before CellTiter-Blue assay. The proliferation assay was performed in triplicate.

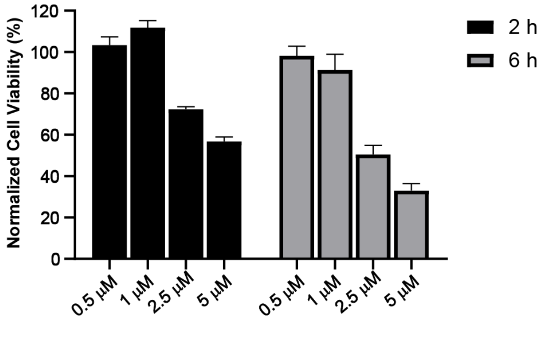

Figure S9. Cytotoxicity of tHCA at different concentrations on MCF-7 cells (n=3; error bars represent the STD).

6.7 Real-time pretargeted tHCA imaging in live cells

MCF-7 cells were seeded at a concentration of 30 000 cells/well in poly-L-lysine coated ibiTreat 8-well μ-slide (ibidi, Germany) and allowed to adhere and adapt to the slide for 36 h. At this point, culture media were changed to complete DMEM with or without 50 μM TZ@SWCNTs. After 6 h treatment, the media were removed. Cellular mitochondria were stained with 250 nM MitoSpy™ Green FM (Biolegend, Cat. 424805) for 20 min and the nuclei were stained with Hoechst 33342 for another 10 min. Then, the cells were rinsed with PBS and cultured with 200 μL of 1 μM tHCA for real time fluorescence imaging using Leica SP5 confocal microscope equipped with a 405 nm (Hoechst 33342), a 488 nm (MitoSpy™ Green FM) and a 633 nm (liberated HCA) laser units through a 63 x/1.4 HCX PL Apo CS Oil objective.

TZ@SWCNTs-Cy3 was used to track the internalization of TZ@SWCNTs. The TZ@SWCNTs-Cy3 was prepared as below: DSPE-PEG-Cy3 were obtained by reacting DSPE-PEG-NHS with Cyanine 3 amine (abcam, ab146462). The TZ@SWCNTs (200 μM) aqueous solution were sonicated with DSPE-PEG-Cy3 (10 μM) for 20 min in water bath sonicator to give TZ@SWCNTs-Cy3. TZ@SWCNTs-Cy3 was washed with Milli-Q water 3 times using centrifugal filter (Amicon Ultra-4, 100 KDa MWCO) to remove the excess DSPE-PEG-TZ and DSPE-PEG-Cy3 before use.

The level of cellular fluorescence in cells treated with tHCA alone or pretargeted strategy was determined using ImageJ-Fiji software by measuring area integrated intensity of cell of interest. For comparison, the intensity from at least 50 cells for each group was measured, averaged and represented as box and whiskers. Statistics were made using unpaired t test with GraphPad Prism 6. Colocalization analysis of MitoSpy™ Green FM and liberated HCA was performed using Coloc2 in ImageJ-Fiji.

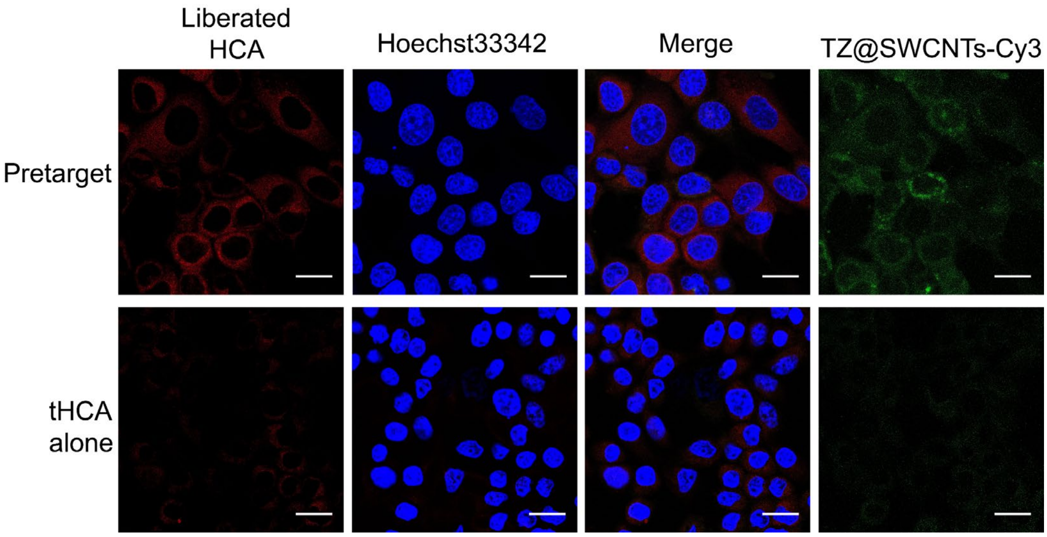

Figure S10. Visualization of TZ@SWCNTs-Cy3 internalization and the pretargeted imaging with tHCA. The images showed the internalization of TZ@SWCNTs (labelled with Cy3) (green) and the liberatation of HCA (red) in pretargeted MCF-7 cells. Scale bar, 20 μm.

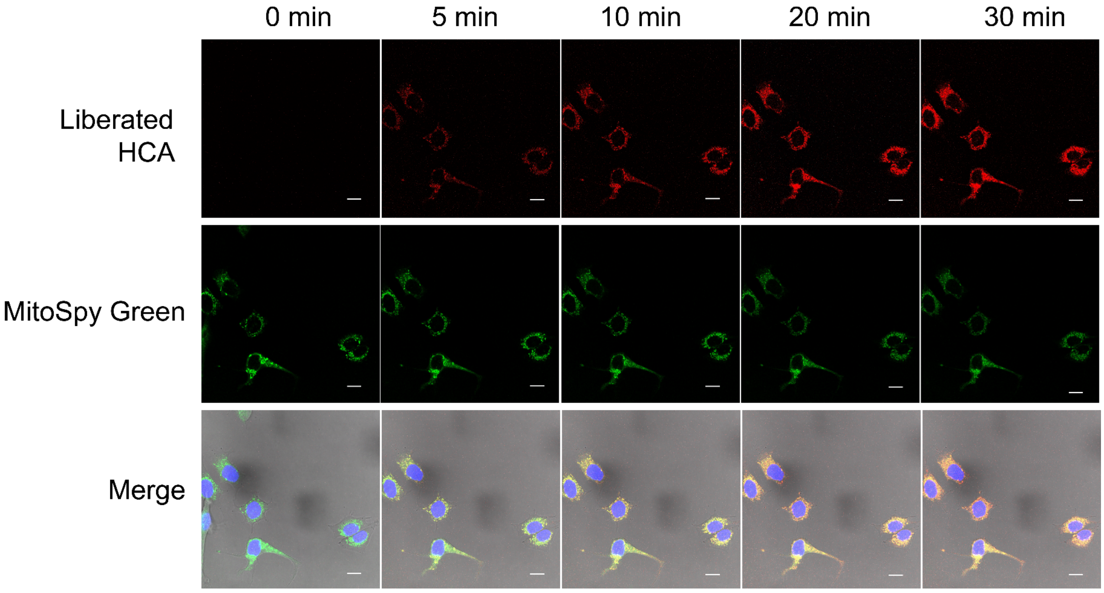

Figure S11. Time-dependent pretargeted tHCA turn-on imaging in MCF-7 cells. Scale bar, 10 μm.

7. Pretargeted fluorogenic imaging in vivo

All animal studies were performed in accordance with the institutional Animal Ethics committee of Instituto de Medicina Molecular and followed the recommendations for care and use of laboratory animals by European commission and Portuguese authorities. Animal experiments were performed under licence “Therapeutic evaluation of anti-cancer conjugates in mouse models of cancer” which was approved by the Direção Geral de Alimentação e Veterinária, Portugal (0421/000/000/216).

Female BALB/c mice (8 –10 weeks old) were purchased from Charles River Laboratories. Tumour-bearing mice were established by subcutaneously injecting a suspension of 0.75E6 CT26 cells in PBS and in Matrigel (ratio 1:1) per mouse into the right flank of each mouse. Mice were randomly assigned to each experimental group (n=5).

TZ@SWCNTs was prepared as described above. TZ@SWCNTs-Cy5 was prepared as below: DSPE-PEG-Cy5 were obtained by reacting DSPE-PEG-NHS with Cyanine5 amine (abcam, ab146463). The TZ@SWCNTs (250 μM) aqueous solution were sonicated with DSPE-PEG-Cy5 (7 μM) for 20 min in water bath sonicator to give TZ@SWCNTs-Cy5. TZ@SWCNTs-Cy5 was washed with water 3 times using centrifugal filter (Amicon Ultra-4, 100 KDa MWCO) to remove the excess DSPE-PEG-TZ and DSPE-PEG-Cy5 before use.

On day 11, mice bearing CT26 tumours were intravenously injected with indicated compounds. For pretargeted strategy, 25 μmol/kg TZ@SWCNTs (corresponding SWCNTs concentration 0.55 mg/kg) in 100 μL PBS was administered. After 2 h interval, a subsequent 2.4 μmol/kg tHCA (60 nmol/mouse) dissolved in 100 μL of 10% mice plasma/PBS was intravenously injected. Saline, TZ@SWCNTs, tHCA alone and TZ@SWCNTs-Cy5 of the corresponding dose and timing were also injected respectively. At indicated time points (1.5, 3, 6 and 24 h) after tail vein injection of tHCA, animals were anesthetized and imaged for whole body NIR fluorescence using an IVIS Lumina fluorescence/bioluminescence imaging system with an excitation filter

of 615 – 665 nm and an emission range of 695 – 770 nm. The images were analysed with Living Image software 3.0 (PerkinElmer).

After the last time point the mice were euthanized, resected and the major tissues and organs were harvested into a 96-well plate for ex vivo fluorescence measurements and assessment of in vivo probe releasing profile using an IVIS Lumina fluorescence/bioluminescence imaging system with an excitation filter of 615 – -665 nm and an emission range of 695 – 770 nm. The images were analysed with Living Image software 3.0 (PerkinElmer).

8. NMR spectra

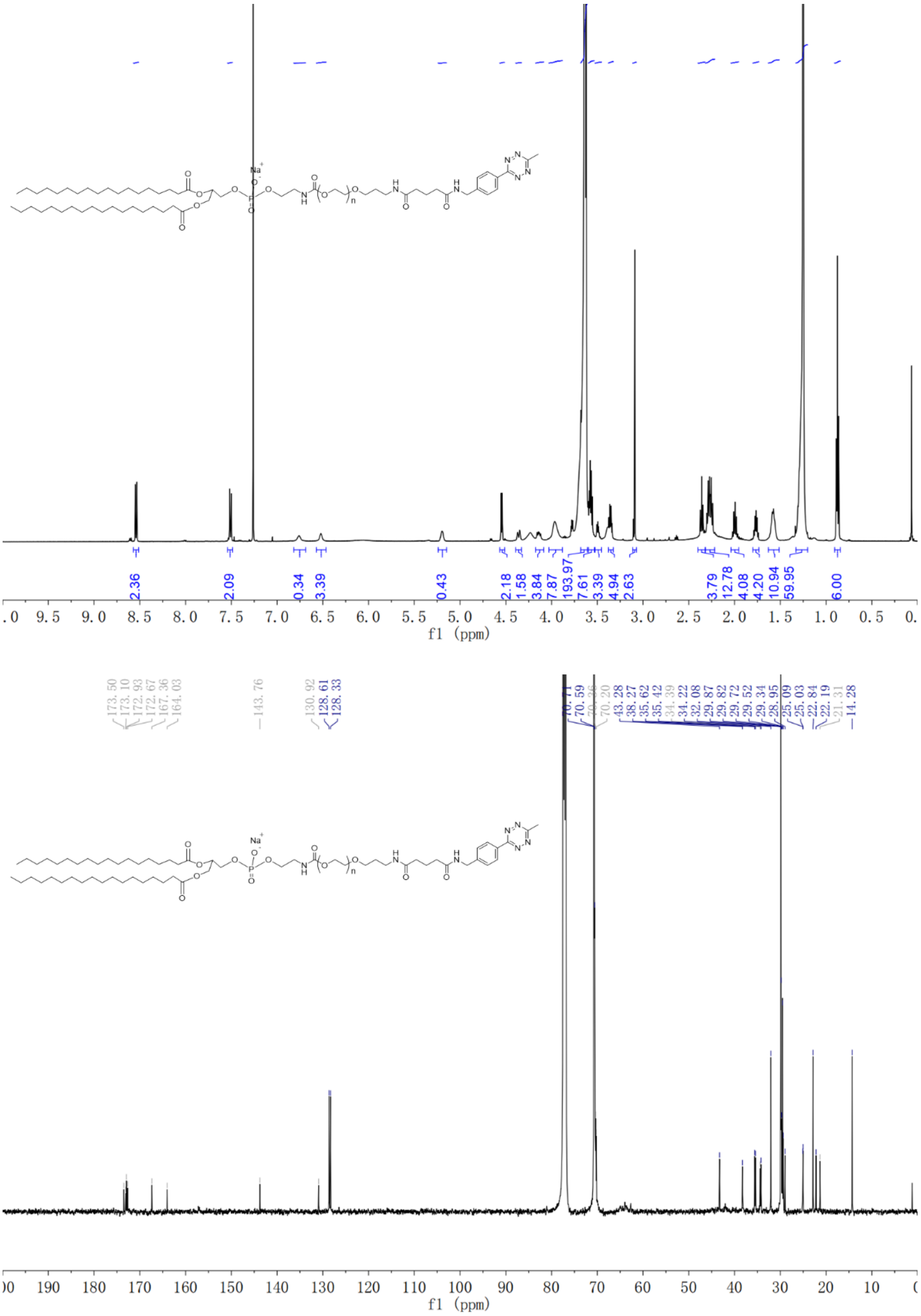

Figure S12. ^{1}H and ^{13}C NMR spectra of DSPE-PEG-TZ.

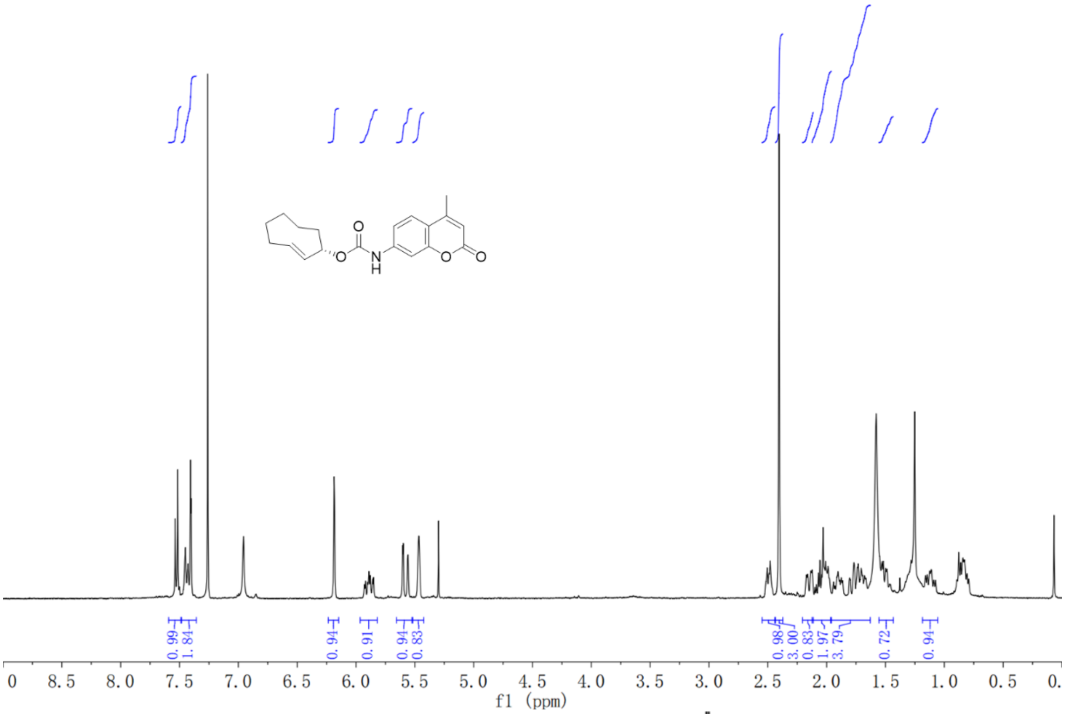

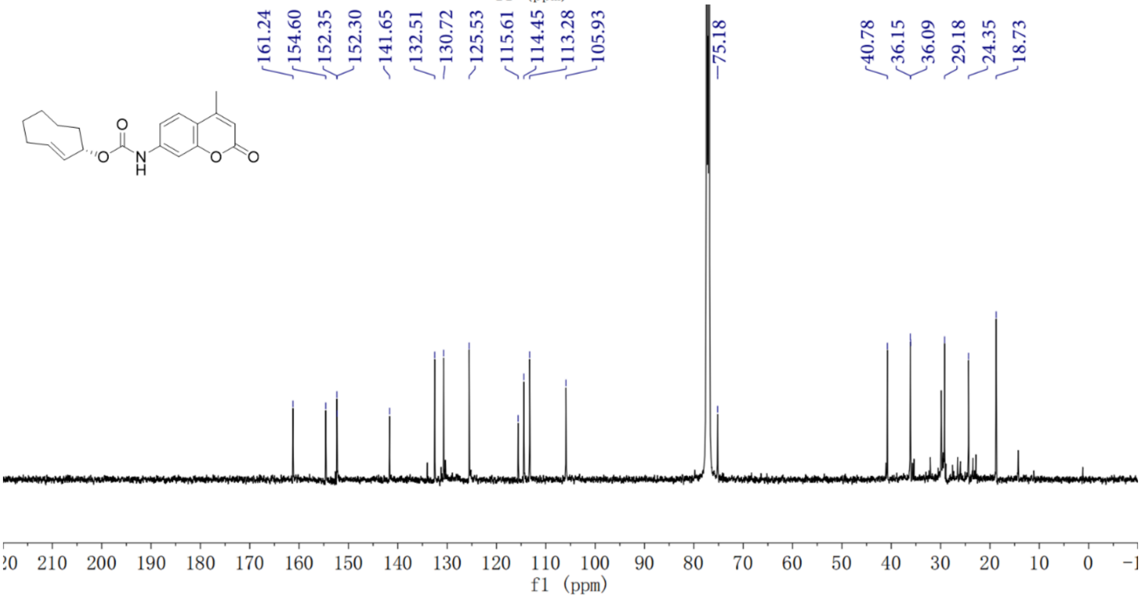

Figure S13. ^{1}H and ^{13}C NMR spectra of tAMC.

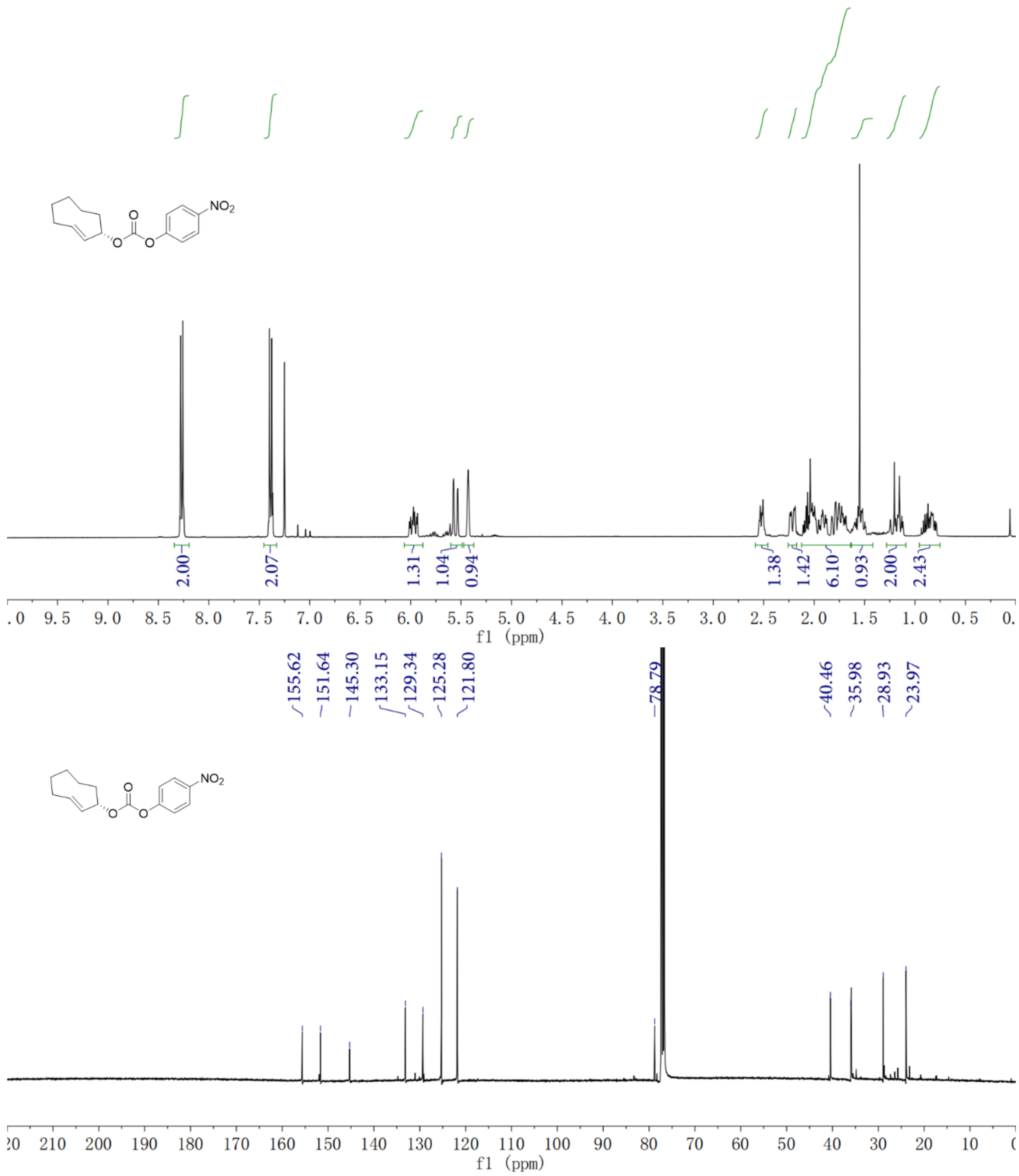

Figure S14. ^{1}H and ^{13}C NMR spectra of (*E*)-cyclooct-2-en-1-yl (4-nitrophenyl) carbonate.

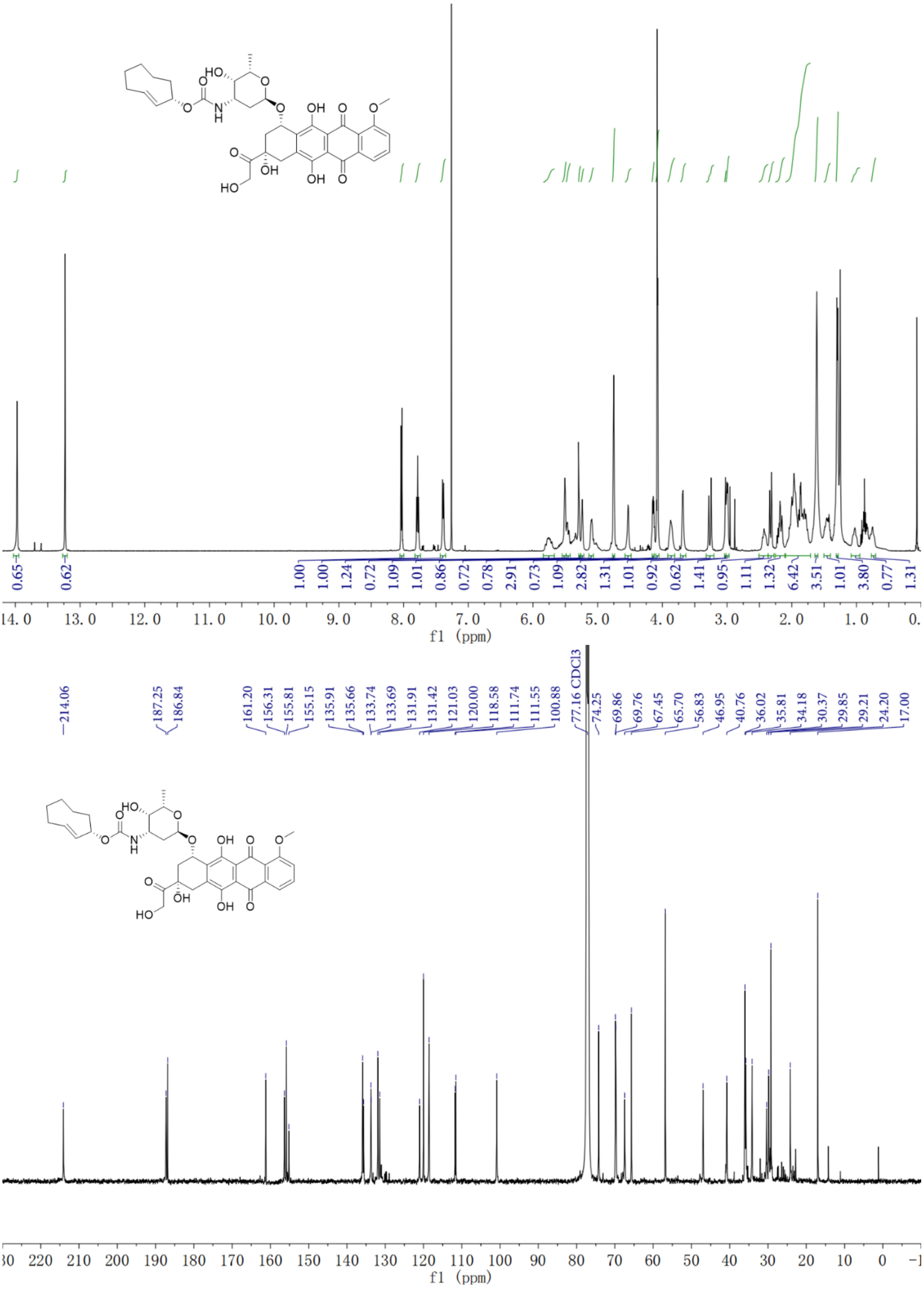

Figure S15. ^{1}H and ^{13}C NMR spectra of tDOX.

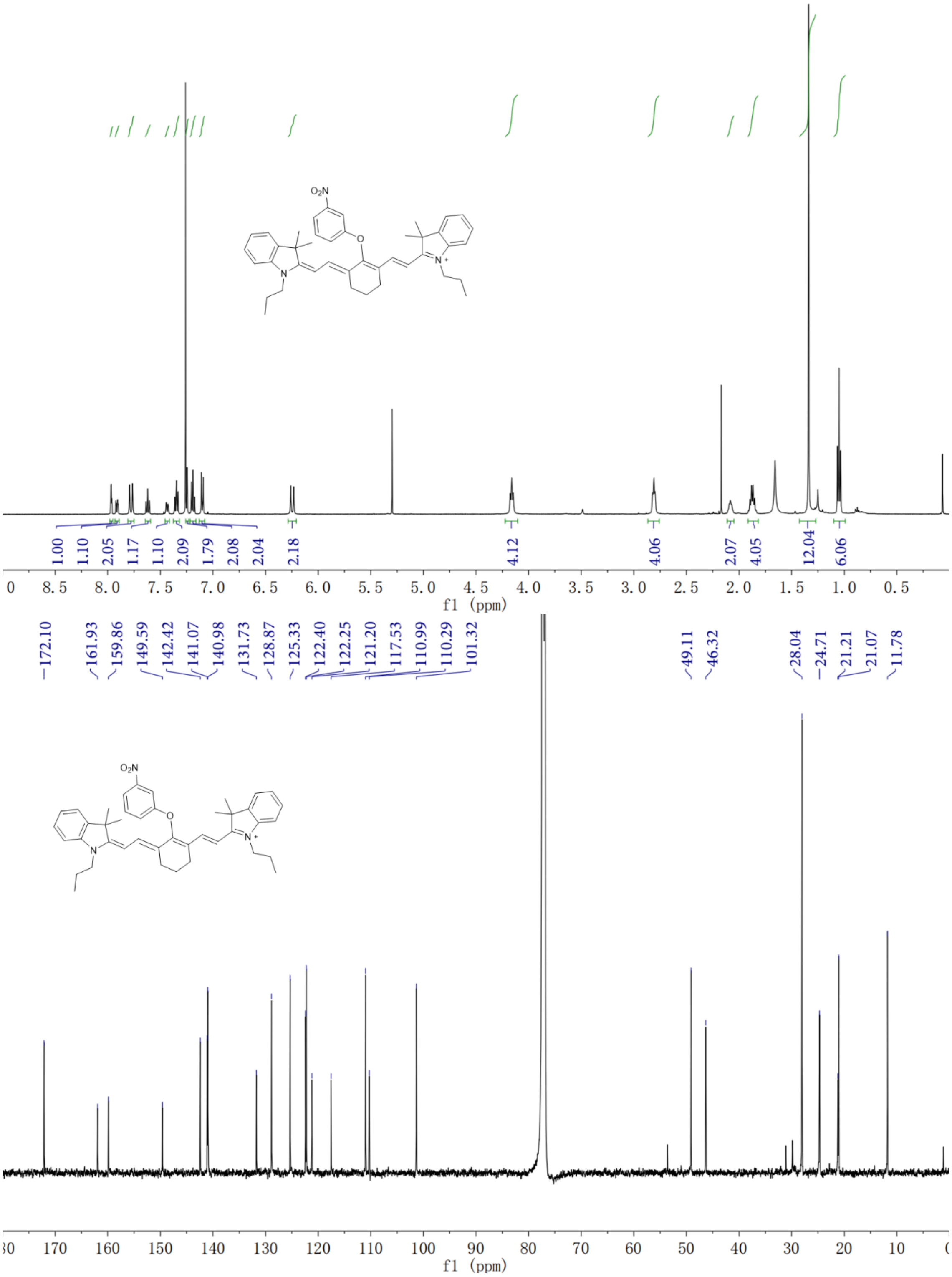

Figure S16. ^{1}H and ^{13}C NMR spectra of IR780-NP.

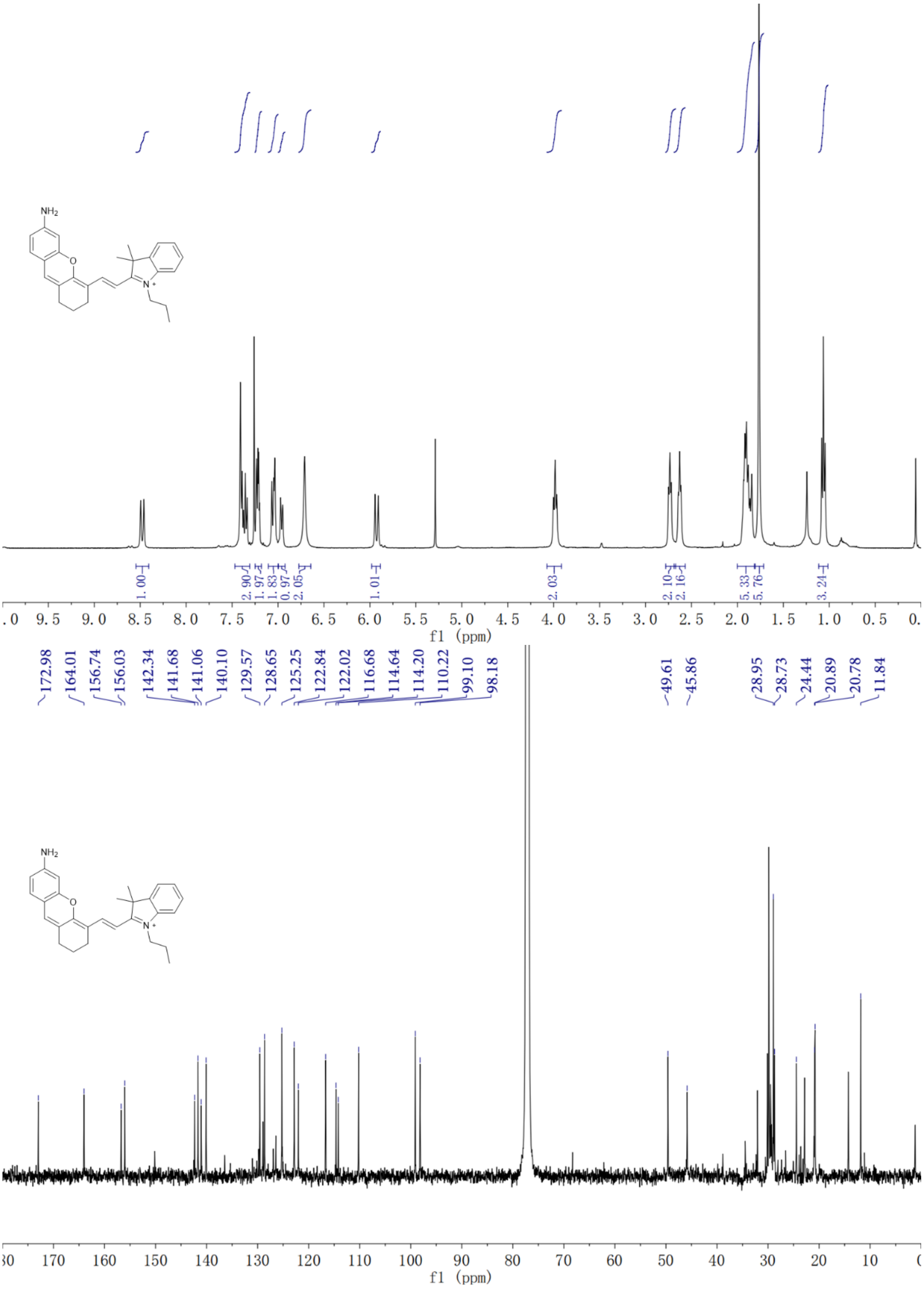

Figure S17. ^{1}H and ^{13}C NMR spectra of HCA.

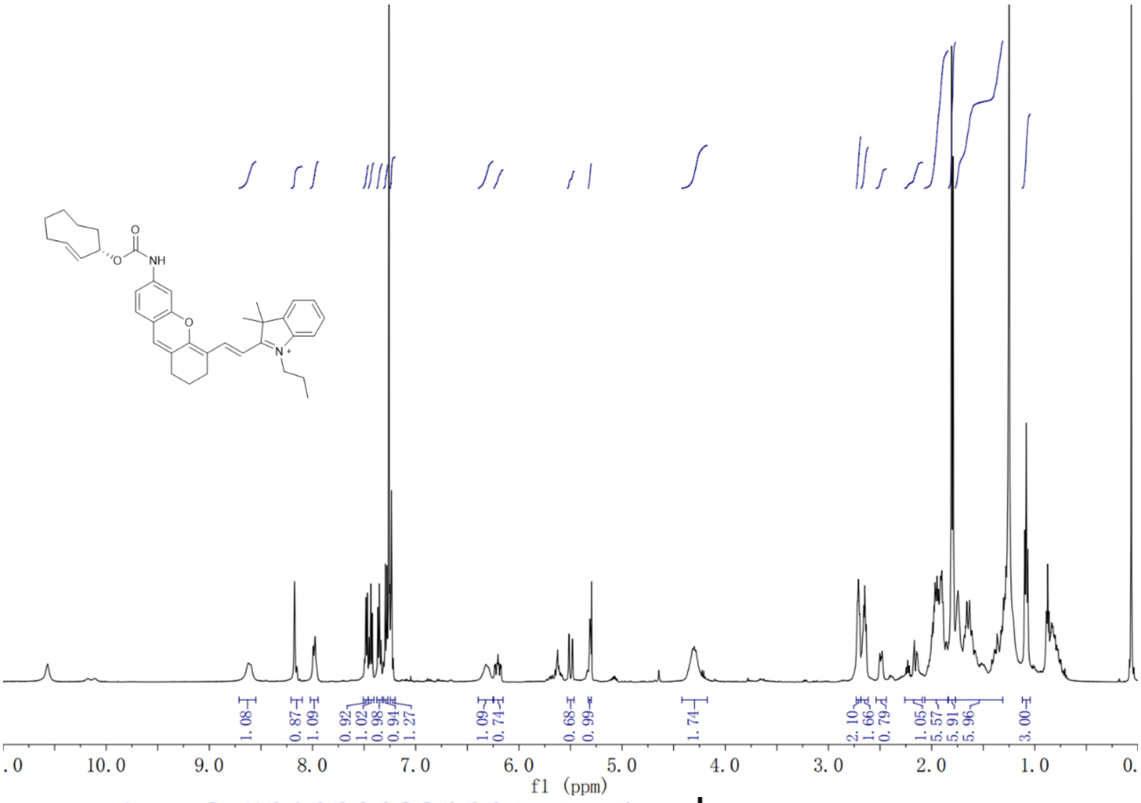

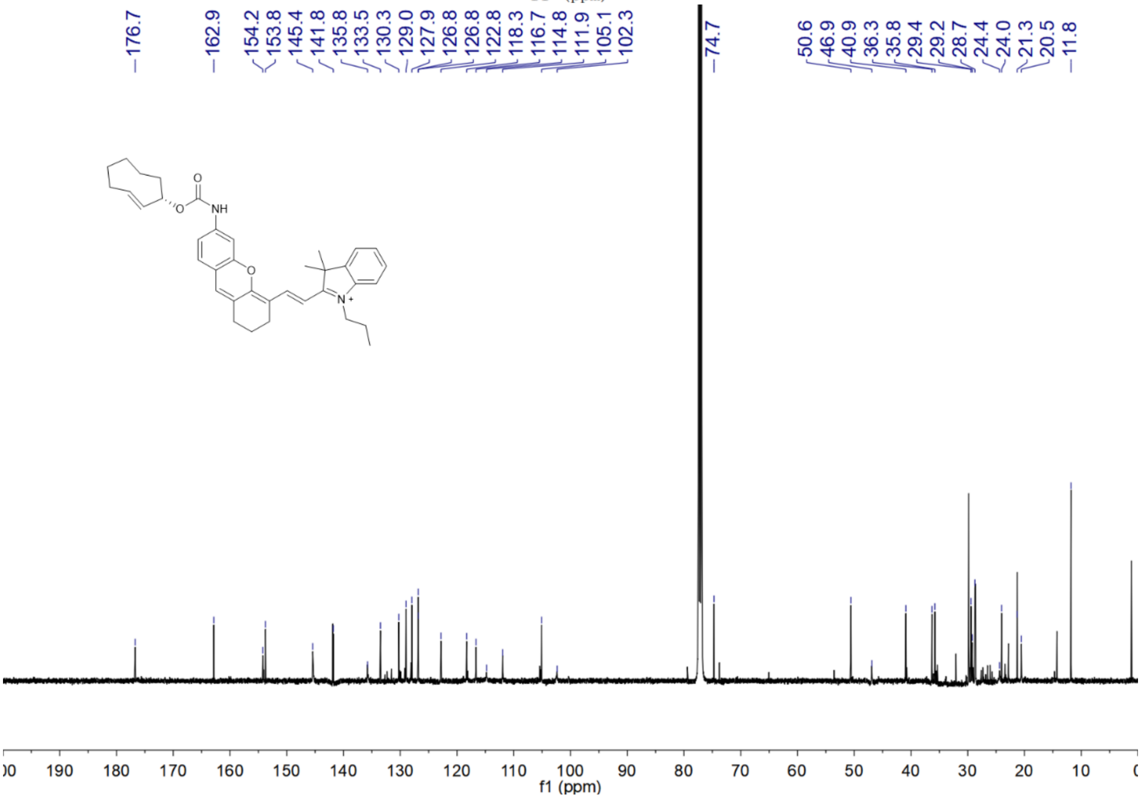

Figure S18. ^{1}H and ^{13}C NMR spectra of tHCA.

9. References

[1] N. W. S. Kam, M. Connell, J. A. Wisdom, H. Dai, *Proc. Natl. Acad. Sci. U. S. A.* **2005**, *102*, 11600–11605.

[2] X. Fan, Y. Ge, F. Lin, Y. Yang, G. Zhang, W. S. C. Ngai, Z. Lin, S. Zheng, J. Wang, J. Zhao, J. Li, P. R. Chen, *Angew. Chem. Int. Ed.* **2016**, *55*, 14046–14050.

[3] R. M. Versteegen, W. ten Hoeve, R. Rossin, M. A. R. de Geus, H. M. Janssen, M. S. Robillard, *Angew. Chem. Int. Ed.* **2018**, *57*, 10494–10499.

[4] a) X. He, Y. Hu, W. Shi, X. Li, H. Ma, *Chem. Commun.* **2017**, *53*, 9438–9441; b) X. He, L. Li, Y. Fang, W. Shi, X. Li, H. Ma, *Chem. Sci.* **2017**, *8*, 3479–3483.
